# p21 as a Transcriptional Co-Repressor of S-Phase and Mitotic Control Genes

**DOI:** 10.1371/journal.pone.0037759

**Published:** 2012-05-25

**Authors:** Nuria Ferrándiz, Juan M. Caraballo, Lucía García-Gutierrez, Vikram Devgan, Manuel Rodriguez-Paredes, M. Carmen Lafita, Gabriel Bretones, Andrea Quintanilla, M. Jose Muñoz-Alonso, Rosa Blanco, Jose C. Reyes, Neus Agell, M. Dolores Delgado, G. Paolo Dotto, Javier León

**Affiliations:** 1 Departamento de Biología Molecular, Instituto de Biomedicina y Biotecnología de Cantabria (IBBTEC), Universidad de Cantabria–CSIC–SODERCAN, Santander, Spain; 2 Cutaneous Biology Research Center, Massachusetts General Hospital, Charlestown, Massachussetts, United States of America; 3 Centro Andaluz de Biología Molecular y Medicina Regenerativa (CABIMER), CSIC, Américo Vespucio s/n, Sevilla, Spain; 4 Instituto de Investigaciones Biomédicas Alberto Sols, CSIC, Madrid, Spain; 5 Departament de Biologia Cellular, Immunologia i Neurociències, Institut d'Investigacions Biomediques August Pi i Sunyer (IDIBAPS), Universitat de Barcelona, Barcelona, Spain; 6 Department of Biochemistry, University of Lausanne, Epalinges, Switzerland; University of Minnesota, United States of America

## Abstract

It has been previously described that p21 functions not only as a CDK inhibitor but also as a transcriptional co-repressor in some systems. To investigate the roles of p21 in transcriptional control, we studied the gene expression changes in two human cell systems. Using a human leukemia cell line (K562) with inducible p21 expression and human primary keratinocytes with adenoviral-mediated p21 expression, we carried out microarray-based gene expression profiling. We found that p21 rapidly and strongly repressed the mRNA levels of a number of genes involved in cell cycle and mitosis. One of the most strongly down-regulated genes was *CCNE2* (cyclin E2 gene). Mutational analysis in K562 cells showed that the N-terminal region of p21 is required for repression of gene expression of *CCNE2* and other genes. Chromatin immunoprecipitation assays indicated that p21 was bound to human *CCNE2* and other p21-repressed genes gene in the vicinity of the transcription start site. Moreover, p21 repressed human CCNE2 promoter-luciferase constructs in K562 cells. Bioinformatic analysis revealed that the CDE motif is present in most of the promoters of the p21-regulated genes. Altogether, the results suggest that p21 exerts a repressive effect on a relevant number of genes controlling S phase and mitosis. Thus, p21 activity as inhibitor of cell cycle progression would be mediated not only by the inhibition of CDKs but also by the transcriptional down-regulation of key genes.

## Introduction

p21^CIP1^ (p21 herein after) is a member of the Cip/Kip family of inhibitors of cell cycle progression (also including p27^KIP1^ and p57^KIP2^). The first discovered p21 function and so far its best studied biochemical activity was the inhibition of cyclin-dependent kinases [1], [2], [3], [4]. CDKs are protein complexes, composed of a regulatory cyclin and a catalytic CDK subunit, which orchestrate cell cycle transitions. Enforced p21 expression results in cell cycle arrest, which frequently takes place at the G2/M transition and accompanied by polyploidy [5], [6], [7], [8]. p21 is also a p53 target gene that plays a relevant role in p53-induced cell cycle arrest [9], [10], [11].

However, other studies have shown that p21 has activities in addition to cell cycle arrest. Thus, p21 acts as an inhibitor of apoptosis induced by DNA-damaging agents [12], [13], [14] and as inducer of senescence [15], [16], [17] or differentiation (reviewed in [18]). Finally, p21 has been implicated in the control of transcription, through mechanisms that may be coupled to its CDK inhibition activity but also by direct association and modulation of transcription factors. In this way, it has been demonstrated the interaction between p21 and several transcription factors such as CBP, C/EBPα, E2Fs, Myc, Nrf2, STAT3, and others (reviewed in [19], [20], [21]). p21 has been found to repress several genes through the interaction with the E2F transcription factor [22] or by other mechanisms [15], [23], [24]. It has also been described as co-activator of the expression of other genes [25], [26], [27], [28]. Nonetheless, there is little information on the biological significance of p21-dependent regulation of gene expression and to what extent it is linked to effects on the cell cycle. It has been shown that CDK2 is not an essential target for p21 in cell cycle inhibition and tumor suppression [29], given further relevance to the gene regulation effects of p21.

To address the relevance of p21-mediated gene regulation we have carried out large-scale expression profiling in two different human systems (keratinocytes and myeloid leukemia cells) upon ectopic expression of p21. p21 provokes a rapid and potent down-regulation of genes involved in the execution and control of mitosis in both models. Mutational analysis revealed that the N-terminal region of p21 is required for its transcriptional effects in leukemia cell. *CCNE2* (cyclin E2) is one of the most potently down-regulated genes and p21 was found to bind and repress the human CCNE2 promoter.

## Methods

### Cell culture, transfection, viral infection

Primary human keratinocytes wereprepared from discarded human tissue, mostly from reduction abdominoplasties, with Institutional Review Board approval (MGH#2000-P-002418/3) and were grown as described [30]. K562 are human chronic myeloid leukemia cells, and were purchased from ATCC. Kp21-4 cells are K562 carrying a Zn^2+^ -inducible p21 gene; Kp27-5 cells are K562 carrying a Zn^2+^ -inducible p27 gene [31]. K562 and derivatives were grown in RPMI with 8% fetal calf serum. Adenovirus infections of keratinocytes were performed for 1 h in serum and epidermal growth factor-free-low calcium medium as previously described [32]. Keratinocytes were then incubated in fully supplemented medium for 24 h prior to collection for RNA analysis. Adenoviruses expressing full-length, N-terminal, C-terminal p21, p16, and p27 have been previously reported [32]. For transient transfections of K562, ten millions of cells were transfected with 12 µg of p21-WT-GFP, p21-CT-GFP and p21-NT-GFP expression vectors, as well as the GFP empty vector [33] using Lipofectamine 2000 (Invitrogene). 12 h after transfection, GFP-expressing cells were harvested by flow cytometry (FACSAria cell sorter, BD Biosciences). For transient transfections of Kp21-4, one million cells were electroporated with 2 µg pLKO-shCDK2 (Open Biosystems) or pLKO.1 using an Amaxa nucleofector (kit V). 24 h after transfection ZnSO_4_ was added (75 µM), the cells were further incubated for 12 h and harvested.

### Flow cytometry

One million cells per sample were fixed with 80% ethanol and stained with propidium iodide as described previously [31]. Cells were analysed by flow cytometry on a FACScant (BD Biosciences). Ten thousand events were gated and analysed using CellQuest software (BD Biosciences).

### siRNA transfection

Human primary keratinocytes were transfected with 200 nM siRNAs for *CDKN1A* gene (p21) (Stealth RNAi, Invitrogen) and control siRNAs (Stealth RNAi negative control, Invitrogen) using Lipofectamine 2000 following the manufacturer's recommendations. 48 h after transfections, cells were analyzed by RT-qPCR (reverse transcriptional-quantitative polymerase chain reaction)

### mRNA determination

Total RNA was isolated using TriReagent (Molecular Research Center) kit (Qiagen). Reverse transcription (RT) was performed with iScript reverse transcriptase (BioRad). Quantitative PCR (qPCR) was performed with the SYBRGreen PCR kit (BioRad) in a BioRad MyiQ apparatus. Primers sequences and amplicon sizes used in the RT-qPCR assays are shown in Table S1. Data were normalized to ribosomal protein S14 (RPS14) mRNA levels.

### Gene expression profiling

Total RNA was prepared using RNeasy kit (Qiagen). Biotinilated cRNA was obtained from total RNA and hybridized to Affymetrix HG-U133A chip in the Genomic Facility of Centro de Investigación del Cancer (Salamanca, Spain). Data analysis and hierarchical tree clusters were generated using the dChip software [34], http://biosun1.harvard.edu/complab/dchip/]. The expression data was filtered so as to include genes with expression changes ≥2.4-fold. The data were obtained in compliance with the MIAME guidelines and are deposited in the ArrayExpress database. The accession numbers are E-MEXP-3431 for the expression data with Kp21-4 and Kp27-5 cells and E-MEXP-3430 for expression data of human keratinocytes. The analysis was performed with data from two independent experiments and RNA preparations of each experimental condition or adenoviral infection. The interaction network for differentially expressed genes was generated with the Ingenuity Pathways Analysis software.

### Immunoblotting and cell extract fractionation

Total cell lysates and immunoblots were carried out as described [31]. Blots were developed with secondary antibodies conjugated to IRDye680 or IRDye800 (Li-Cor Biosciences) and visualized in an Odyssey scanner. Antibodies used were anti-p21 (C-19), anti-UBF (F5), anti-CDK2 (M2) and anti-α-tubulin (H-300). All were polyclonal antibodies from Santa Cruz Biotech. The isolation of chromatin fraction was performed essentially as described [35]. Briefly, the cells were lysed for 20 min with agitation at 4°C in CSK buffer (10 mM HEPES pH 7.5, 100 mM NaCl, 300 mM sucrose, 3 mM MgCl_2_, 0.5% Tritón X-100 and protease inhibitors). After centrifugation the supernatant was collected as cytoplasmic plus nucleoplasmic fraction. The pellet was washed at maximum setting with CSK buffer, resuspended in CSK buffer, sonicated and saved as chromatin fraction.

### Chromatin Immunoprecipitation (ChIP) assays

ChIP analysis was carried out as described previously [36]. Briefly, 10 millions of K562, Kp21-4 and Kp27-5 cells were fixed with formaldehyde and lysed in SDS lysis buffer. Cross-linked chromatin was fragmented by sonication to an average size of 400 bp. Chromatin was then immunoprecipitated with anti-p21 (C-19, Santa Cruz Biotechnology), anti-p27 (C-19, Santa Cruz Biotechnology), anti-CDK2 (M2, Santa Cruz Biotechnology), anti-histone H3 (FL-136, Santa Cruz Biotecnology) and anti-acetylated-histone H3 (06-559, Millipore). Antibodies and cell lysates were incubated overnight at 4°C, and then with protein G-coupled magnetic beads (Dynabeads, Invitrogen) for 1 h at 4°C. Controls were performed by incubating parallel samples with non-immune IgG. The protein-DNA cross-links were reversed by 4 h incubation at 65°C, and immunoprecipitated DNA was analyzed by quantitative PCR using a BioRad MyiQ apparatus. The signals were normalized to the inputs and the signals obtained with normal rabbit IgG (Santa Cruz Biotechnology) The primers used for PCR reactions are indicated Table S1.

### Luciferase reporter assays

Two million of cells were electroporated with 15 µg of pCyCE1-Luc [37], [38] carrying a fragment of the human CCNE1 and CCNE2 promoters, respectively, upstream of the firefly luciferase. These luciferase reporters were co-transfected with pCEFL or pCEFL-p21 [39]. Cells were electroporated with the reporters and expression vectors (15 µg) at 260 v and 975 µFa in a BTX electroporator. 24 h alter transfection the cells were treated with 75 µM ZnSO_4_ for another 24 h. Luciferase activity was then determined with Lysis Solution 1 (Promega), Luciferase Substrate (Promega) and a GloMax 20/20 luminometer (Promega), following the manufacturer's instructions. All transfections were normalized by measuring β-galactosidase activity of the samples. Data are the average of at least three independent experiments and error bars indicate standard deviation.

## Results

### p21 represses mitotic genes in human leukemia cells

**Figure 1:**
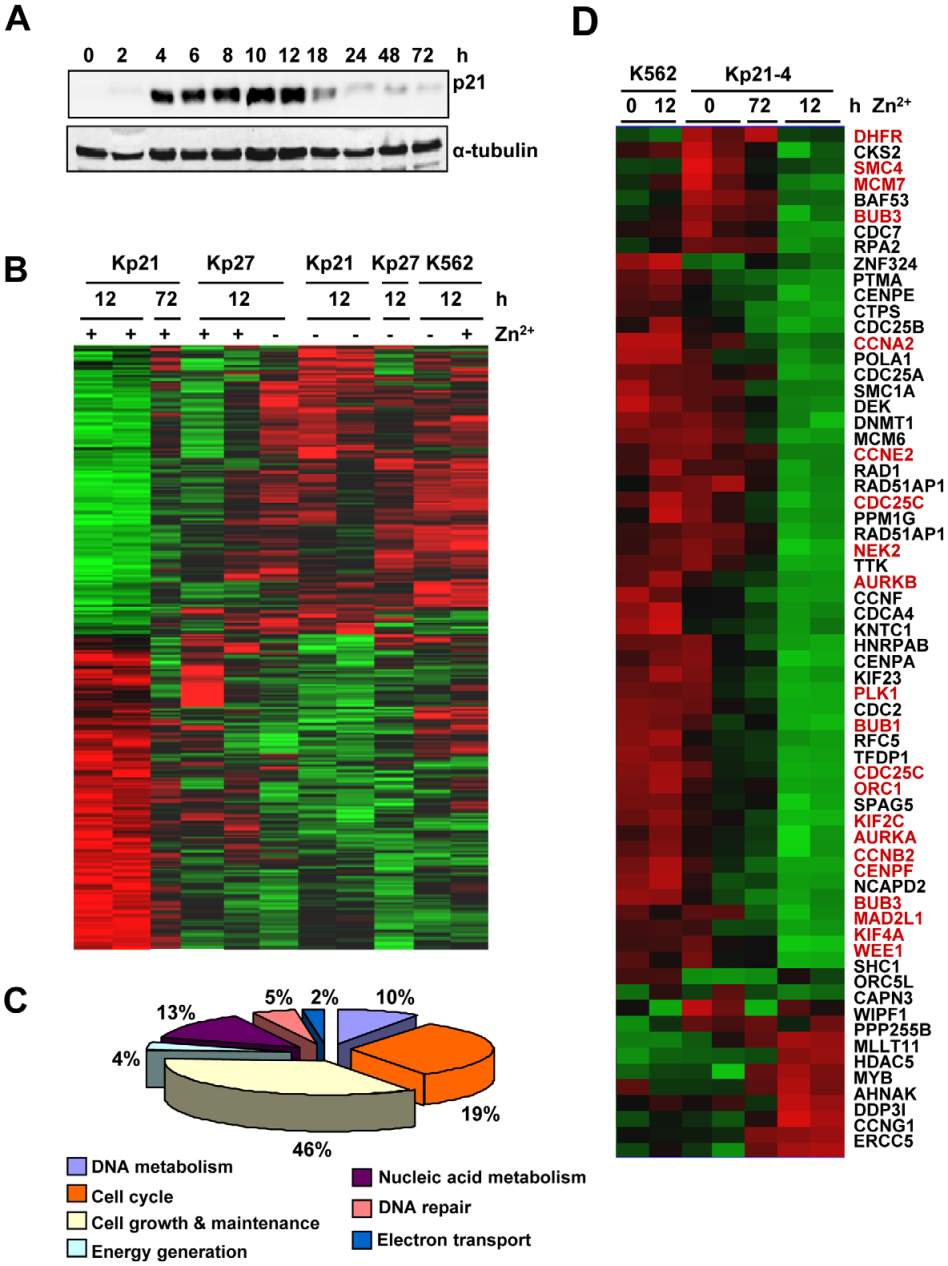
Gene expression changes induced by p21 in K562 cells. A. Expression of p21 in Kp21-4 cells in response to ZnSO_4_. Cells were treated with 75 µM ZnSO_4_ for the periods of time indicated. Cell extracts were prepared and the p21 levels analyzed by immunoblot. B. The transcriptome of Kp21-4 cells (Kp21) treated for 12 h with ZnSO_4_ (to induce p21) were compared to that of cells with induced p27 (Kp27), parental K562 and Kp21-4 treated for 72 h with ZnSO_4_. The heat map shows the hierarchical clustering with those genes with expression variation ≥2.3-fold between uninduced and p21-induced Kp21-4 cells (P<0.001). The heat map shows 350 regulated genes (360 gene probes). C. Distribution of the regulated genes shown in A according with their cell functions. The ontogeny analysis has been carried out with the dChip program. D. Expression changes in 65 genes related to cell cycle and mitosis according to the ontogenic classification. The heat map was obtained as described in B. The genes further validated by RT-qPCR (Figure 2A) are shown in red.

In order to find genes regulated by p21 in human primary cells we carried out a gene expression profiling in human myeloid leukemia K562 cells with conditional expression of p21. We previously described a K562 derivative, termed Kp21-4, that carries a zinc-inducible p21 gene [31]. We performed a kinetic study to identify the expression peak of p21 in this system. The immunoblot results showed that treatment of Kp21-4 cells with 75 µM ZnSO_4_ resulted in induction of p21 that peaked 4–12 h decreasing afterwards (Figure 1A). This transient induction of p21 was accompanied by proliferation arrest and an increase in polyploid cells after 48–72 h [31]. The cell cycle profile did not change over the first 12 h of p21 induction with ZnSO_4_ but 6–12 h of p21 induction were sufficient to irreversibly trigger proliferation arrest and polyploidy (Figure S1). Therefore, we chose 12 h as the induction time to analyse p21 effects on the transcriptome of these cells, as gene expression changes later on may be indirect due to other phenotypic effects.

We next carried out the gene expression profiling of Kp21-4 cells upon p21 induction by ZnSO_4_. In order to identify genes specifically modulated by p21 we compared with the cell line Kp27-5, which carries a Zn^2+^-inducible p27 allele [31]. p27 is a close relative to p21 that also inhibits CDKs and induce cell cycle arrest [40]. Thus, the comparison serves to identify genes specifically regulated by p21 in our analysis. We subtracted the gene expression changes occurring at 72 h in Kp21-4 cells those genes regulated by p27 in the Kp27-5 cells and genes changed by ZnSO_4_ treatment in parental K562 cells. We found 350 genes whose expression changed ≥2.3 fold in Kp21-4 after 12 h of p21 induction and which were not regulated at this time point in Kp27-5 or K562 cells treated with ZnSO_4_ (Figure 1B). The list of the genes with their corresponding expression change is shown in the Table S2.

**Figure 2:**
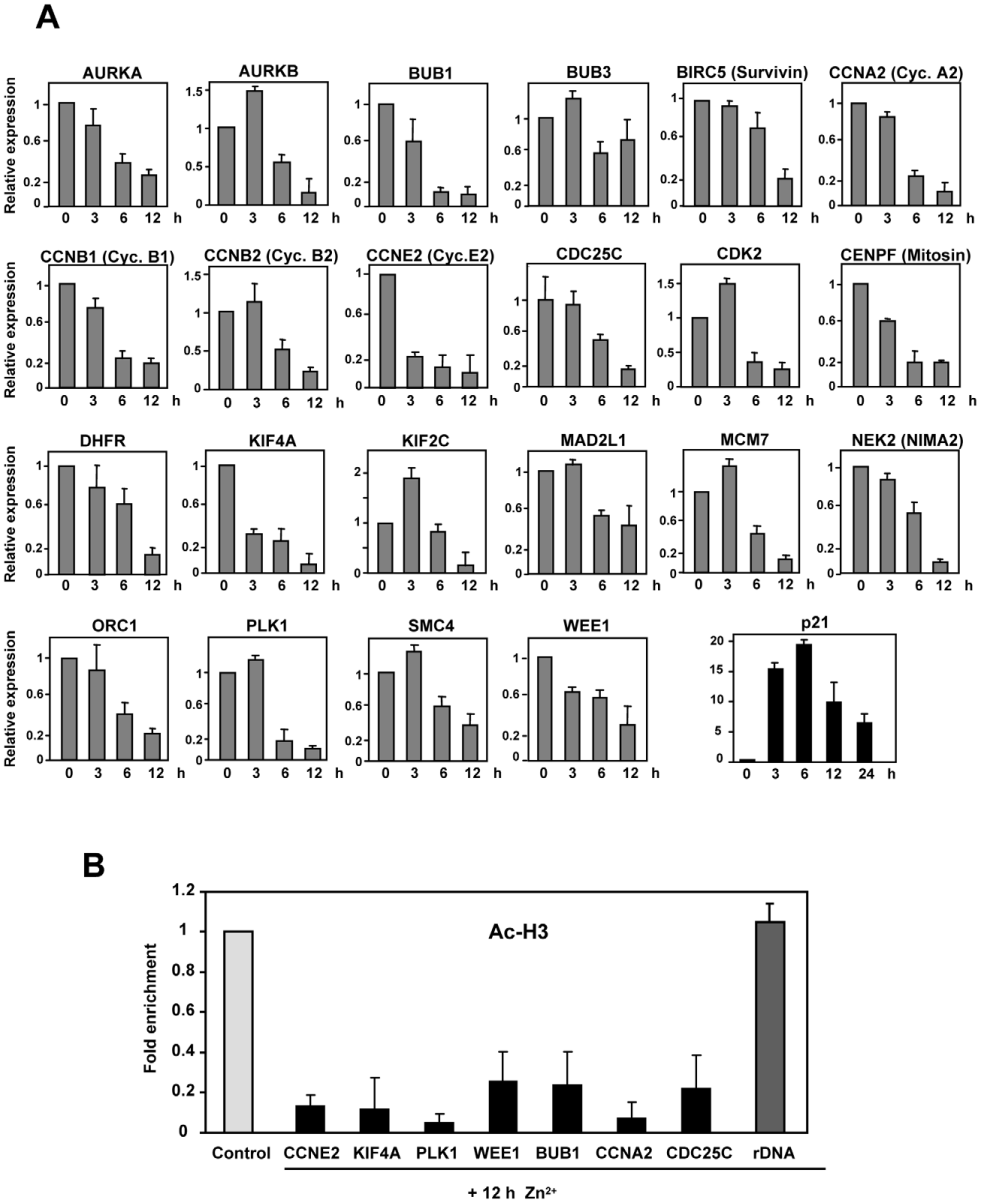
p21-mediated the down-regulation of genes involved in cell cycle. A.The expression of p21 was induced in Kp21-4 cells by 75 µM ZnSO_4_ and 3, 6 and 12 h later, total RNA was prepared and expression of the indicated genes was determined by RT-qPCR. In some cases an alternative name is given into brackets. Cyc., cyclin. The values are means ±S.E.M. from two independent experiments and two determinations for each RNA. B. Kp21-4 cells were treated for 6 h with 75 µM ZnSO_4_ to induce p21. Chromatin immunoprecipitation was carried out with anti-histone H3 and anti-acetylated-histone H3 antibodies and (as specificity control) rabbit IgG. The DNA in the immunoprecipitated chromatin was measured by quantitative PCR. The amplicons encompass the transcription start site of the indicated genes. A regulatory sequence of the promoter of rDNA was used as a control. The results are expressed as the ratio of DNA enrichment in chromatin immunoprecipitated with anti-H3 versus acetylated-H3 (“Ac-H3”) and normalized to the values obtained in uninduced cells. The values are the means ±S.E.M of two independent ChIP experiments with two PCR determinations each.

The dataset used for the clustering analysis of Figure 1B was further analyzed with the Ingenuity Pathways software to reveal the network of interactions between differentially regulated genes. The results showed that the two highest-ranked networks were assembled by interactions between genes related to cell cycle control (Figure S2). Gene ontology analysis revealed that cell cycle-related genes accounted for about one fifth of the regulated genes, i.e., 65 genes (Figure 1C). The expression change of these genes is shown as a heat map in Figure 1D, demonstrating that the vast majority of these genes were down-regulated by p21. The former result defined cell cycle and mitosis as the most relevant functional categories of p21-down-regulated genes. We investigated the kinetics of changes in the RNA levels of 19 genes repressed by p21 from those appearing in Figure 1D plus *BIRC5*, *CCNB1* and *CDK2*, because of their involvement in cell cycle. RNA was prepared from Kp21-4 cells and expression was determined by RT-qPCR at different periods of time up to 12 h after ZnSO_4_ addition, i.e., when p21 expression is maximal and its effect is already irreversible. The results are shown in Fig. 2A and confirmed that most of the down-regulated genes identified by microarray hybridization were down-regulated as soon as 6 h after the addition of the p21 inducer. ZnSO_4_ did not modify the expression of any of these genes in parental K562 (data not shown). To confirm the repressive effect of p21 we analyzed the level of acetylated-histone H3, a marker of active chromatin [41], in the chromatin region corresponding to the transcriptional start site of several genes. The results showed a dramatic decrease in the fraction of acetylated-histone H3 as soon as 6 h after p21 induction (Fig. 2B). The data also argues that the decrease in mRNA levels is caused by transcriptional switch-off rather than mRNA degradation

**Figure 3:**
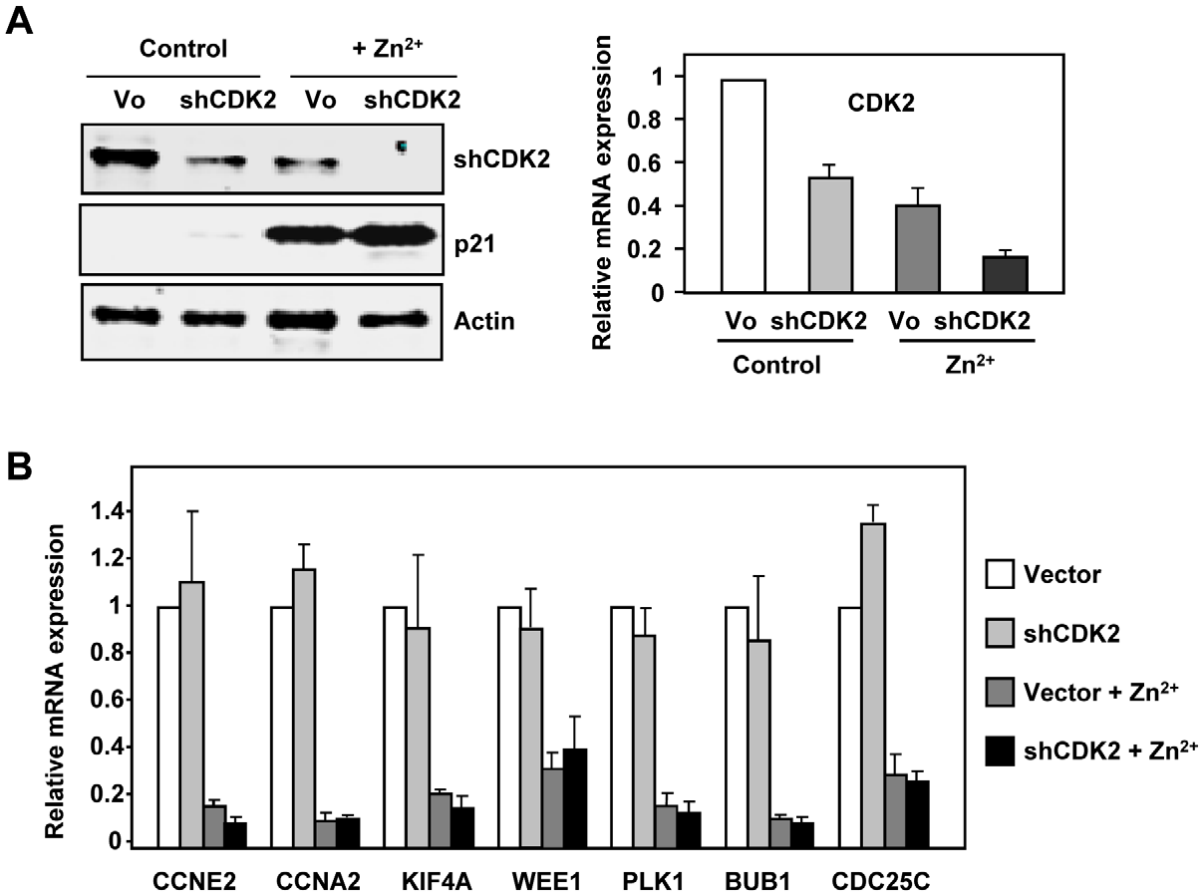
p21-mediated repression of genes is not dependent on CDK2. A. K562 cells were transiently transfected with a short-hairpin CDK2 (“shCDK2”) vector and the empty vector (pLKO.1, “Vo”). 24 h after transfection the cells were treated with 75 µM ZnSO_4_ for 12 h to induce p21 and the silencing of CDK2 was assayed at the protein level by immunoblot (left panel) and at the mRNA level by RT-qPCR (right panel) The expression of p21 and actin was also determined to control the p21 induction by Zn^2+^ and the protein level, respectively. B. The expression of p21 was induced with 75 µM ZnSO_4_ in K562 transfected with sh-CDK2 and 12 h later, total RNA was prepared and expression of the indicated genes was determined by RT-qPCR. The values are means ±S.E.M. from two independent experiments and two determinations for each RNA.

The fast kinetics of gene down-regulation caused by p21, before any change in cell cycle profile could be detected, suggested that the repression is a direct consequence of p21 activity, rather than an indirect effect. CDK2 inhibition is the best known biochemical activity of p21, and it also occurs in K562 [31]. Thus, we explored the possibility that the p21-dependent gene expression regulation described above is a consequence of CDK2 inhibition. We conducted two sets of experiments. First, we showed that the depletion of CDK2 in Kp21-4 cells achieved through siRNA did not modify the p21-dependent repression of the assayed genes. This has been demonstrated by transient expression of a short-hairpin CDK2 vector (Fig. 3). Second, we analysed the effects of p27, which also provokes CDK2 inhibition and cell cycle arrest [31], on the expression of genes down-regulated by p21. In contrast to p21, p27 provoked a dramatic G1 arrest already detectable after 6 h of induction in Kp27-5 cells (Figure S1). p27 was induced in Kp27-5 cells for 3–12 h and the expression of 11 genes was assayed by RT-qPCR. The results showed that p27 did not repress cell cycle genes or repressed them with a much slower kinetics than p21 (Figure S3), despite a similar induction level of p21 and p27 in p21-4 and Kp27-5 cells respectively (Figure S1A). Moreover, we performed gene expression profiling in Kp27-5 cells upon 12 h of p27 induction. The results showed that at this induction time p27 elicited weaker effects on gene regulation than p21. After subtraction of p21-regulated genes the analysis revealed that p27 regulated only 180 genes (with an expression change ≥2.3 fold after 12 h of p27 induction) and none of them was annotated as related to cell cycle, according to the Gene Ontology analysis (Figure S4). The comparison to retrieve shared gene expression profiles by p21 and p27 induction revealed 90 common genes that were regulated by both proteins after 12 h of induction. These genes belong to different functional categories, but only 8 genes were related to cell cycle (Figure S4).

### The N-terminal region of p21 is sufficient for gene down-regulation

**Figure 4:**
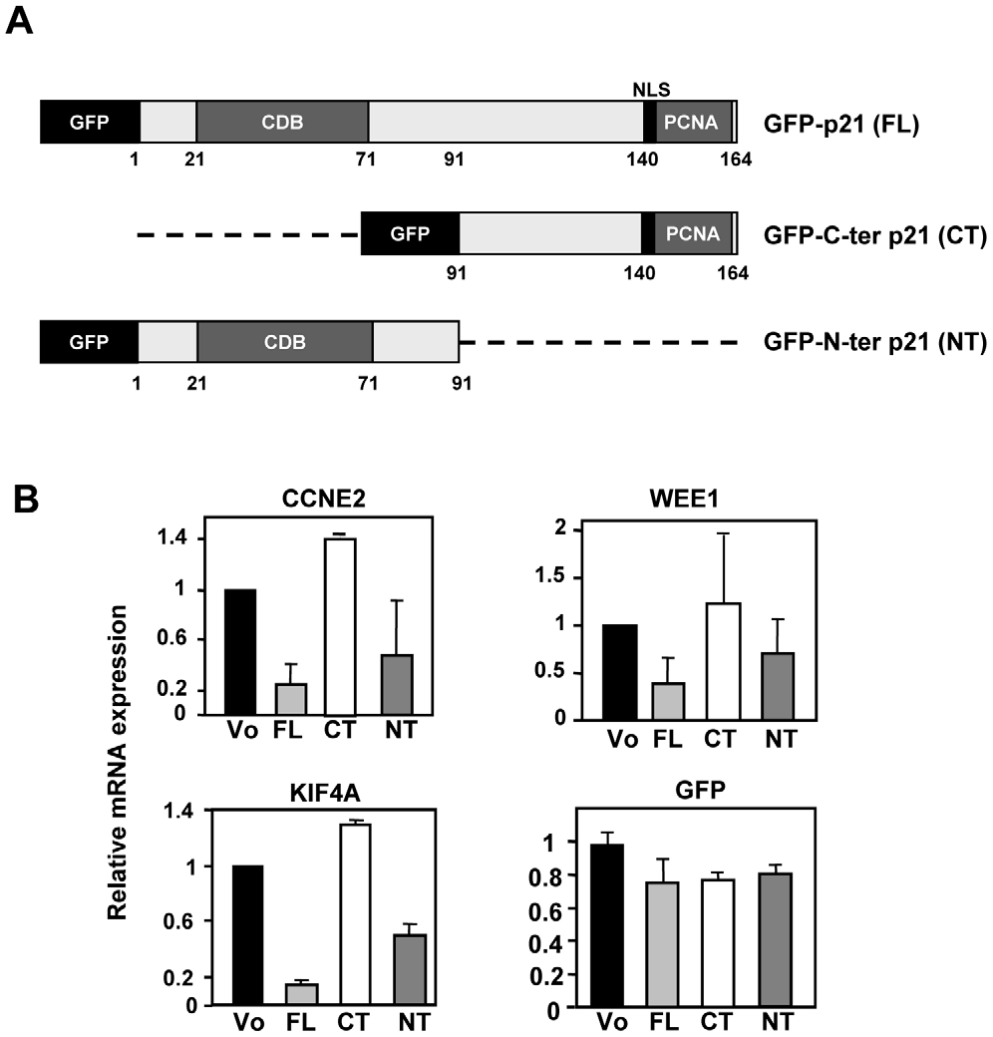
The N-terminal region of p21 is required for its effect as gene repressor. A. Schematic representation of the p21 constructs used. The proteins carry the green fluorescent protein (GFP) in the N-terminal. The position of the CDK binding domain (CDB) and the PCNA binding domain (PCNA) are indicated. B. K562 cells were transfected with expression vectors for the full-length p21 protein (FL), the p21 amino-terminal region (NT) and the p21 carboxy-terminal region (CT). 24 h after transfection the cells were sorted by flow cytometry and the expression of the indicated genes was analysed by RT-qPCR. The values are means ±S.E. from two transfections and two determinations of each mRNA.

Next, we asked whether the CDK-cyclin binding domain of p21 was involved in the down-regulation of gene expression. We used two p21 deletion constructs carrying the green fluorescent protein. One construct carried the first 91 amino acids, which included the CDK-cyclin binding region and the second construct the C-terminal region, after the codon 91 [33] (Figure 4A). Although the GFP-N-terminal construct lacked the nuclear localization signal in the p21 region, it partly localizes in the cell nuclei, likely due to the GFP domain [33]. We transfected these constructs as well as the full-length p21-GFP construct and selected the GFP-expressing cells by fluorescent-activated cell sorting. We further tested the expression of several genes with rapid (*CCNE2*, *KIF4A*) and slower (*WEE1*) down-regulation kinetics (see Figure 2). The expression of *CCNE2*, *WEE1* and *KIF4A* in GFP-positive cells was determined by RT-qPCR in the transfected cells. The results showed that the N-terminal p21 was sufficient to provoke the gene down-regulation, although it was a less efficient repressor than the full-length p21. In contrast, C-terminal p21 was inactive as repressor of the assayed genes (Figure 4B).

This indicates that the repressive activity of p21 is independent of the protein-protein interaction domain known to reside in the C-terminal region, as PCNA binding [42].

### p21 binds to the human CCNE2 promoter

**Figure 5:**
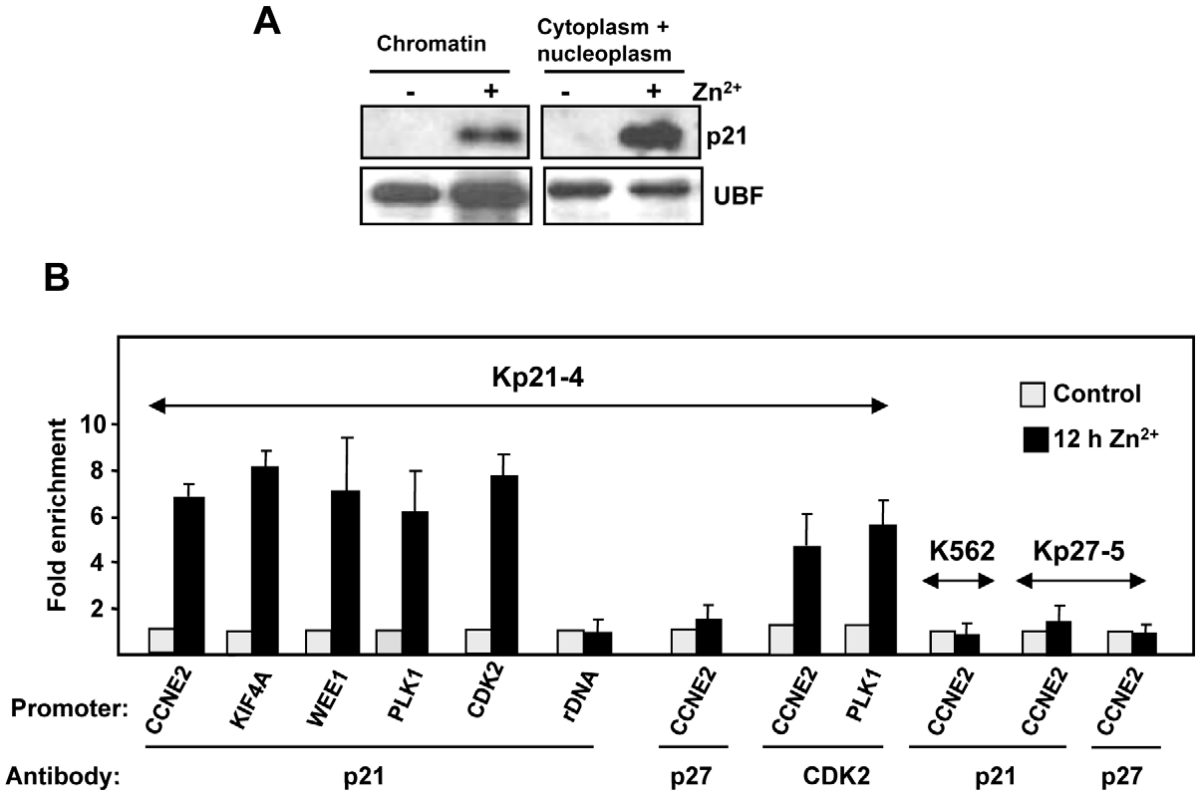
p21 binds to human CCNE2 promoter. A. Kp21-4 cells were treated for 12 h with ZnSO_4_ and the nuclear extracts were fractionated between insoluble (chromatin) fraction and soluble (nucleoplasmic) fraction. The p21 levels were determined by immunoblot. The transcription factor UBF was used as control of chromatin-bound protein. B. Cells were treated for 12 h ZnSO_4_ to induce p21 (in Kp21-4 cells), p27 (in Kp27-5 cells), parental K562 cells were also treated with ZnSO4 as negative control. Chromatin immunoprecipitation was performed with anti-p21, anti-p27 and anti-CDK2 antibodies as indicated, and (as specificity control) rabbit IgG. The DNA in the immunoprecipitated chromatin was analysed by quantitative PCR. The amplicons encompass the transcription start site of the indicated genes. rRNA promoter sequences were used negative controls. The results are expressed as enrichment of DNA in chromatin immunoprecipitated with anti-p21 (with respect the signal with anti-rabbit IgG) in cells induced with Zn^2+^, and normalized to the values obtained in uninduced cells. The values are the means ±S.E.M of three PCR determinations, each from two independent ChIP experiments.

The observation that p21 provokes a rapid mRNA repression of various genes, suggested that p21 might participate in gene repression as a transcriptional modulator, a function already reported for p21 (see Introduction). For further analysis, we focused on the *CCNE2* gene because of its pivotal role in S phase entry and because it showed one of the fastest down-regulation kinetics, with a decrease as soon as 6 h after Zn^2+^ addition (Figure 2). We previously reported that most p21 remains nuclear upon induction with Zn^2+^ in Kp21-4 cells [43]. However, in order to act as co-regulator it is required that at least part of the p21 induced by Zn^2+^ in Kp21-4 cells is bound to chromatin. We first carried out a fractionation of Kp21-4 nuclear extracts into chromatin and nucleoplasmic fractions. The results of the immunoblot show a significant amount of p21 bound to the chromatin fraction in Kp21-4 cells induced with ZnSO_4_ (Figure 5A). This result led us to ask whether p21 could be bound to the promoter of their regulated genes. We performed chromatin immunoprecipitation (ChIP) in Kp21-4 cells with anti-p21 antibody, and asked for p21 binding in the vicinity of the transcription start site (TSS) of human *CCNE2* and other p21-repressed genes. The results showed that p21 was bound to the region encompassing the TSS in Kp21-4 cells upon p21 induction after 12 h of treatment with ZnSO_4_ (Figure 4B). We also found p21 binding to the promoters of other p21-target genes as *CDK2*, *KIF4A*, *PLK1* and *WEE1* to similar extent than to *CCNE2* promoter (Figure 5B). As additional controls, we performed ChIP experiments in Kp27-5 cells treated with ZnSO_4_ to induce p27 [31] and in parental K562 also treated with ZnSO_4_. No enrichment was found in the chromatin precipitated with anti-p21 in both cases. Also, no specific signal was detected with anti-p27 antibody in Kp21-4 cells and in Kp27-5 cells with induced p27 expression (Figure 5B). As the p21 region required for repression includes the cyclin-CDK binding region (Figure 4) we also performed ChIP assays with anti-CDK2 antibody. The results show that in cells overexpressing p21, CDK2 is recruited to the same site of human *CCNE2* that p21 (Figure 4B), which is in concordance with the presence of p21 on that region of the chromatin.

### p21 represses CCNE2 promoter activity

**Figure 6:**
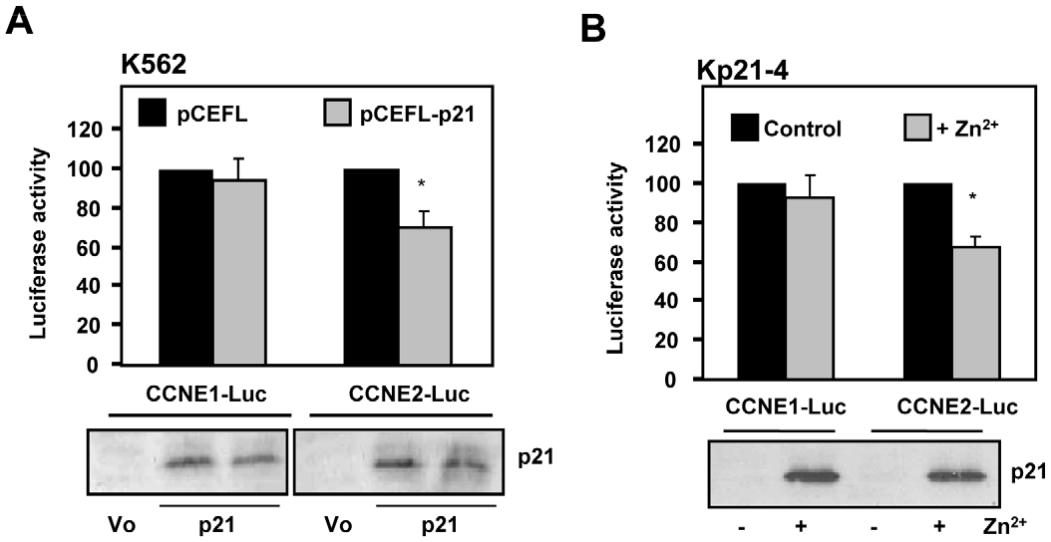
p21 represses the activity of human CCNE2 promoter. A. K562 cells were transfected with luciferase reporters carrying the promoters cyclin E1 and E2 genes (*CCNE1* and *CCNE2*), along with an expression vector for p21 and the corresponding empty vector, and a beta-gal plasmid for transfection efficiency normalization. 24 h after transfection the luciferase activities were determined. The data are normalized to the activity of cells transfected with the empty vector. Lower panel: immunoblot analysis of the transfected cells to assess the expression of p21. B. Kp21-4 cells were transfected with luciferase reporters carrying the promoters cyclin E1 and E2 genes as in (A) and 24 h later the cells were treated with 75 µM ZnSO_4_ and after 24 h the luciferase activities were determined. The data are normalized to the activity of cells without ZnSO_4_. Lower panel: immunoblot analysis of the transfected cells to assess the expression of p21.

The former results show that CCNE2 is rapidly down-regulated by p21 and that p21 is bound to CCNE2 proximal promoter in Kp21-4 cells. To confirm the hypothesis that p21 may act as a transcriptional modulator we carried out luciferase assays with reporters harbouring the human *CCNE2* and (as a control) *CCNE1* promoters. The results demonstrated a modest but significant and reproducible decrease in *CCNE2* promoter upon transfection of a p21 expression vector (Figure 6A). As a second approach, we next used the Kp21-4 cell line and performed the luciferase assays 24 h after the induction of p21 (Figure 6B). Immunoblot analysis revealed the overexpression of p21 in transfected cells (Figure 6A and 6B, lower panels). Taken collectively the results strongly suggest a role of p21 in the negative regulation of *CCNE2* and other genes in the K562 system.

### p21 represses mitotic genes in human primary keratinocytes

**Figure 7:**
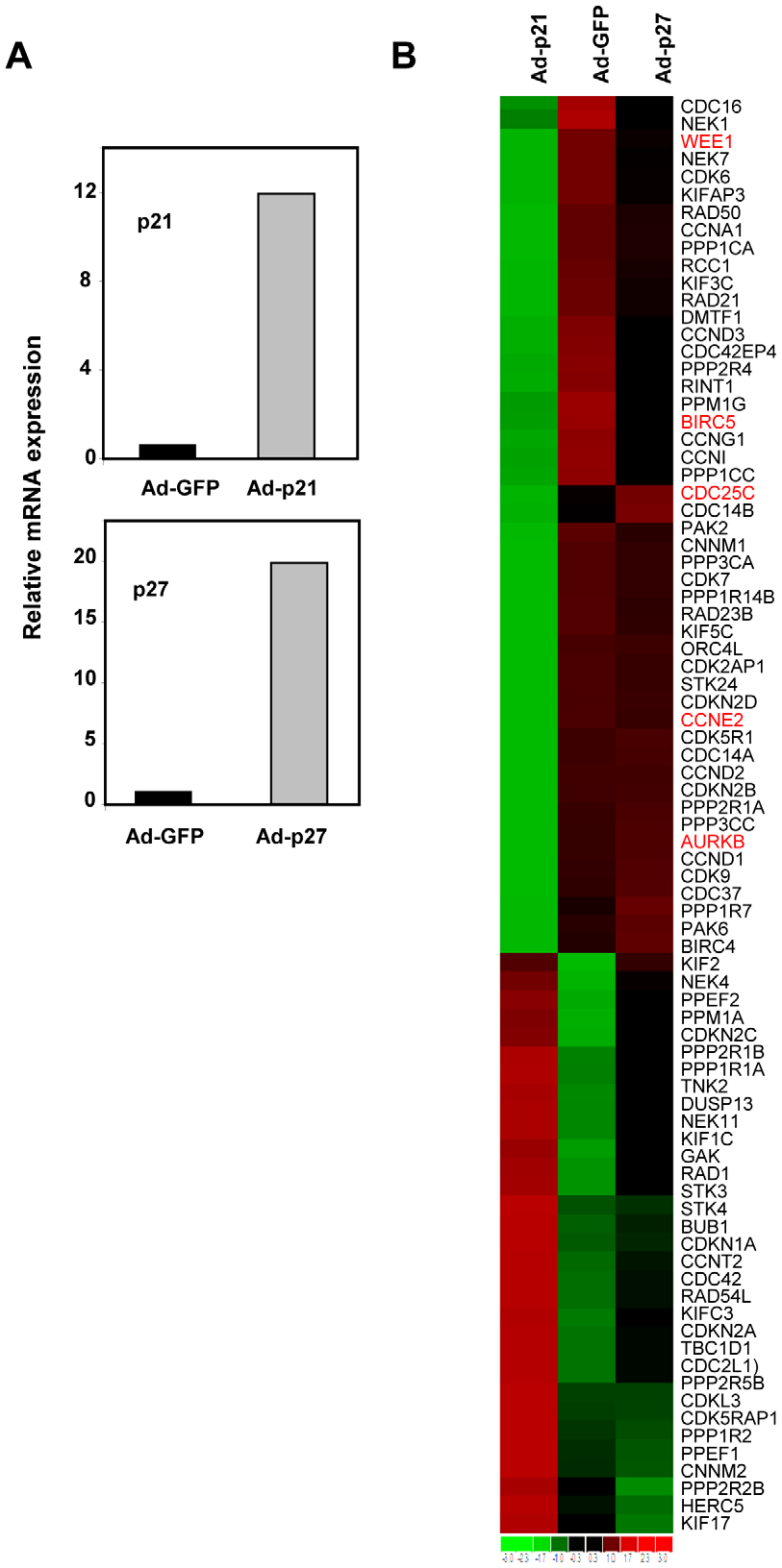
Genes regulated by p21 in human keratinocytes related to cell cycle and cell division. A. Primary human keratinocytes were infected with recombinant adenoviruses expressing p21 (Ad-p21), p27 (Ad-p27). As a control, cells were also infected with adenovirus expressing GFP (Ad-GFP). The mRNA expression of p21 (upper graph) and p27 (lower graph) was determined by RT-qPCR 24 h after infection. Data are represented relative to the expression in Ad-GFP-infected cell. B. Heat-map showing the 82 genes changed by p21 related to cell cycle and cell division with an expression change > 2.3-fold. The names of the regulated genes are indicated at the right. Green indicates genes down-regulated by p21 and red genes up-regulated.

**Figure 8:**
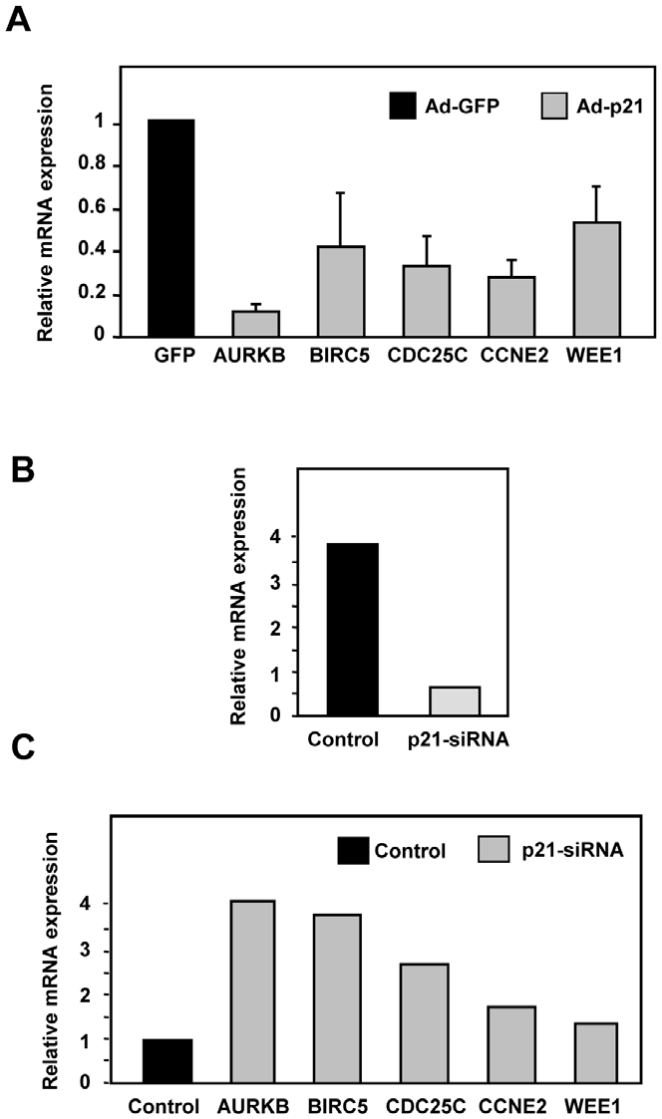
p21-mediated down-regulation of genes involved in mitotic control in human keratinocytes. A. Primary human keratinocytes were infected with adenovirus expressing p21 and ad-GFP as a control. 24 h after infection total RNA was prepared and the mRNA levels of the indicated genes were analysed by RT-qPCR. The values are means ±S.E. from three determinations. B. keratinocytes were transfected with siRNA for p21 and a negative control siRNA. Total RNA was prepared and the mRNA levels of the indicated genes were analysed by RT-qPCR. C. Primary human keratinocytes were transfected with p21 siRNA or control siRNA. 48 h after lipofection total RNA was prepared and the mRNA levels of the indicated genes were analysed by RT-qPCR.

All the previous results have been obtained in a cell line derived from human myeloid leukemia. In order to confirm these results we studied the p21-dependent repression of mitotic genes in a different cellular system. We chose human primary keratinocytes because they are non-tumorigenic, non-immortalized and epithelial cells, in contrast to K562 cells. Human primary keratinocytes were infected with recombinant adenoviruses expressing the full-length p21 protein. A dramatic increase in p21 in infected keratinocytes was demonstrated by RT-qPCR (Figure 7A). As controls, we also infected the keratinocytes with adenovirus carrying the genes for p27, which overexpression was also confirmed by RT-qPCR (Figure 7A). We prepared RNA 24 h after infection and performed large-scale expression assay using the Afftymetrix platform. The clustering analysis revealed that p21 provoked the down-regulation of a number genes involved in cell cycle control not shared by cells expressing p27 (Figure 7B). The list of the genes with their corresponding expression change is shown in the Table S3. We next validated by RT-qPCR the p21-mediated repression of several of these genes involved at various checkpoints of cell cycle (*AURKB*, *BIRC5*, *CDC25C*, *CCNE2* and *WEE1*). The results demonstrated the decreased levels of mRNA for all the tested genes, confirming the data of the microarray hybridization (Figure 8A). To fully confirm the effect of p21 as a repressor of these genes in keratinocytes, we silenced p21 through siRNA transfection (Figure 8B). The expression of the five genes was tested in p21-depleted cells and we found that the five genes were up-regulated to various extents (Figure 8C). The results therefore confirm the regulation of the expression of the analyzed genes by p21.

### Bioinformatic analysis of p21-targeted genes

**Figure 9:**
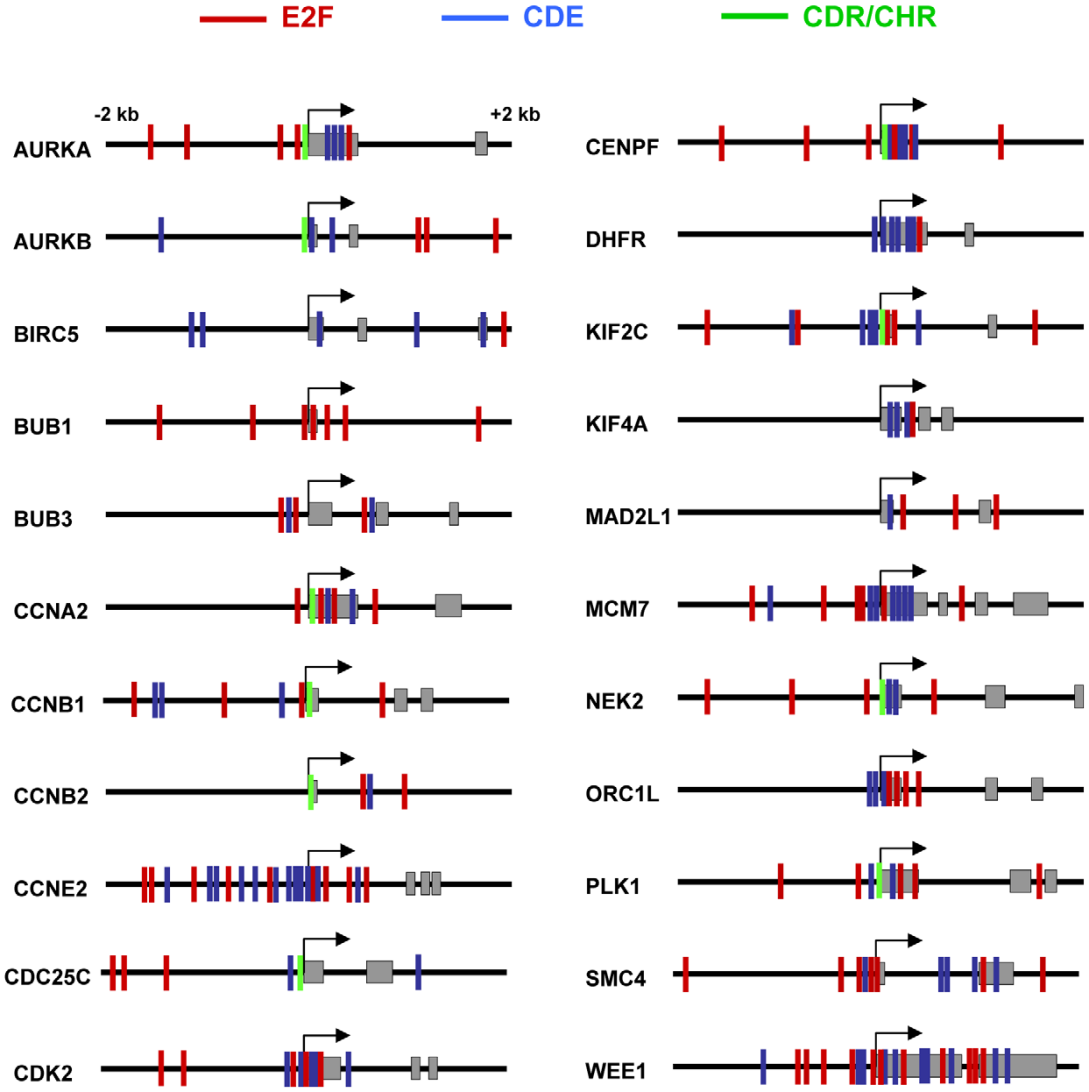
Putative regulatory sites in genes down-regulated by p21. Schematic representation of the E2F (red), CDE (blue) and CDE/CHR (green) sites in the p21-regulated genes analysed in Figure 2. The region analysed encompass 2 kb upstream and downstream the transcription start site (marked by an arrow). Exons are represented as grey boxes. E2F sites: TTT^C^/_G_^C^/_G_^C^/_G_^C^/_G_. CDE sites: ^T^/_G_GGCGG. CDE/CHR sites: GCG^C^/_G_N_2–5_ TT^A^/_G_AA.

As noted in the Introduction, it has been reported that p21 binds to specific DNA sequences of some p21-regulated genes. As p21 lacks any recognizable DNA-binding domain, the interaction must be indirect, i.e., through another transcription factor or co-factor. We previously reported that p21 can bind genes at E2F-binding elements [23]. Another report showed the binding of p21 to the CDE/CDH element, composed by the the cell cycle-dependent element (CDE) and the cell cycle genes homology region (CHR) [44]. Therefore, we have performed a bioinformatic analysis of the promoters of the 22 genes where p21-mediated down-regulation was validated by RT-qPCR (shown in Figure 2). The results show that a majority of the genes carried several E2F sites in the 5′ regulatory region and/or the first exon (Figure 9) but there were some exceptions (*BIRC5*, *CCNB2*, *DHFR*, *KIF4A*). More interestingly, all genes but one (*BUB1*) contained at least one CDE site in the vicinity of the TSS (Figure 9). In this context it must be noted that human *CCNE2* promoter and first exon are particularly rich in CDE sequences. Thus, p21 may impair transcription of genes in charge of the execution and control of mitosis through the interaction with transcription factors binding to CDE or CDE/CHR motifs.

## Discussion

Despite the original description of p21 as a CDK inhibitor, a number of reports have described a role of p21 as transcriptional modulator for different genes such as *WNT* [23], cyclin D1 [27], polo-like kinase 1 (*PLK1*), topoisomerase IIα [44], *CDC25A*, *MYC* [24] and p53 [45]. p21 has also been described as an antagonist of E2F-dependent transcription [22]. Conversely to previous studies, we have analyzed the genome-wide p21-dependent gene regulation in two different human cellular models: a myeloid leukemia cell line (K562) and primary keratinocytes. p21 overexpression was attained through the induction of a conditional transgene by ZnSO_4_ (in human myeloid cells) or through acute adenoviral infection (in human keratinocytes).

Our results on the gene expression profiling in both cell models showed that p21 provokes a rapid repression of mRNA of genes involved in cell cycle and mitosis control in the two models. Since p21 elicits cell growth arrest this effect could be indirect, i.e., a consequence of the cell cycle arrest. However, in myeloid cells with inducible p21 (Kp21-4 cells), the kinetics of the mRNA down-regulation of most of the analysed genes was almost parallel to the kinetics of the p21 protein up-regulation. This down-regulation of mRNA was also concomitant with the rapid deacetylation of histone H3 at the TSS. The genes repressed by p21 were up-regulated upon p21 silencing, confirming the p21 activity. Moreover, the early p21-induced down-regulation was not reproduced by p27 induction in K562 cells form most genes, despite that p27 also provokes a cell cycle arrest via CDK inhibition. Furthermore, the depletion of CDK2 does not affect the p21-mediated gene repression. Altogether these data argue against the idea that the p21 effects were a direct consequence of the CDK inhibition or of the cell cycle arrest. Thus, it is conceivable that p21 is directly involved in transcriptional repression of a set of genes involved in cell cycle control. Previous unsuccessful attempts to generate a functional p21 fused to the estrogen receptor (so as to get activated by tamoxifen) [46] precluded the analysis of p21 transcriptional effects in the absence of protein synthesis.

It is remarkable the similarity in the short-term transcriptional effects of p21 overexpression observed in two very different cell types (keratinocytes and myeloid cells), despite the different long-term consequence of p21 overexpression in each cell type. p21 inhibits epidermal differentiation in keratinocytes [25], [32] whereas it induces polyploidy and megakaryocytic differentiation in K562 [31]. It is of note however, that the transcriptional changes that we have observed occur before any phenotypic change can be detected in either cell line. In Kp21-4 cells, the polyploidy is detectable 3–4 days after induction and at this time point, the expression of CDC25, cyclin A and cyclin E are recovered, likely to allow DNA synthesis for endoreplication [8]. Previous studies in a fibrosarcoma cell line also showed p21 repressed genes involved in mitosis followed by polyploidy three days after p21 induction [7], suggesting that polyploidization may be a common effect of p21 in tumor cells.

We further analysed the regulation of *CCNE2* (cyclin E2), one of the genes undergoing a fastest down-regulation. ChIP assays revealed that p21 binds in the vicinity of the TSS of human cyclin E2 gene (as well as other four genes: *CDK2*, *KIF4A*, *PLK1* and *WEE1*). The results are consistent with previous literature showing chromatin binding of p21 in several models (see Introduction). In contrast, upon induction of p27 for 12 h in Kp27-5 cells, we could not detect significant binding of p27 to CCNE2 promoter.

In full agreement with the results observed at the mRNA level and ChIP assays, luciferase reporter experiments showed that p21 repressed the human *CCNE2* promoter. The mutational analysis revealed that CCNE2 gene repression depends on the N-terminal region of p21, i.e., the region involved in the binding to cyclin-CDK complexes. The C-terminal domain of p21 is not required, but seems to contribute for full activity of p21 as repressor. It is noteworthy that p21 does not repress CCNE1 promoter (if it does) as efficiently as CCNE2, arguing for a differential role of both cyclins.

Taken together the data argue for a role of p21 as co-repressor of gene transcription for genes related to cell cycle. As p21 lacks any recognizable DNA-binding domain, the interaction must be indirect, i.e., through another transcription factor or co-factor. It has been reported that p21 binds to specific DNA sequences of some genes. For instance, we have previously shown that this is the case for *WNT4* repression, which depends on the interaction with E2F1 [23]. Also, it has been reported that p21 repress the mitotic control gene PLK1 through binding to the CDE/CHR element [44]. The CDE/CHR elements control the transcription of genes with maximum expression in G2 phase and in mitosis and are repressed in G0 and G1 phases, although the transcription factor(s) responsible are still unidentified [47]. It has been recently reported that p27 binds to some promoters through E2F4 sites in mouse fibroblasts [48]. Our analysis showed that many of the p21-downregulated genes contained one or several E2F binding sites, but at least four genes did not (*BIRC5*, *CCNB2*, *DHFR*, *KIF4A*), despite that a relaxed consensus sequence as E2F's binding site was used for the analysis, which includes E2F4 sites (Figure 9). Our analysis does not give information regarding the specific E2F family member involved, so the differential binding of E2F factors on the different genes is an open possibility. More interestingly, we found that all genes but one (*BUB1*) contained at least one CDE site in the vicinity of the TSS. In this context it must be noted that human CCNE2 promoter and first exon are particularly rich in CDE sequences. Thus, p21 may impair transcription of genes in charge of the execution and control of mitosis through the interaction with transcription factors binding to CDE or CDE/CHR motifs.

Altogether, our results indicate that p21 is a multifunctional protein with the capacity to act through at least two mechanisms to control cell cycle: directly inhibiting CDKs and indirectly regulating genes involved in cell cycle control. The relative importance of each mechanism await further investigation, but it is of note that p21 is able to arrest the cell cycle in CDK2-deficient cells [29], arguing for the importance of transcriptional repression in the p21 functions in cell biology. Additional studies are required to dissect out the mechanisms involved in the transcriptional repression mediated by p21.

## Supporting Information

Figure S1 Cell cycle alterations mediated by p21 and p27 in K562 cells. A. Immunoblots showing the induction of p21 in Kp21-4 cells and p27 in Kp7-5 cells after 6 and 12 h of treatment with 75 µM ZnSO_4_. B. Absence of cell cycle profile alteration after short induction times of p21. Cell cycle profile of Kp21-4 and Kp27-5 cells upon induction of p21 and p27 with 75 µM ZnSO_4_ for 6 and 12 h. The cell cycle profile was determined by flow cytometry of propidium iodide-stained cells. C. p21 induces an irreversible accumulation of G2 and polyploid cells whereas p27 induces a reversible accumulation in G1. Kp21-4 and Kp27-5 were treated with ZnSO4 for 12 h. The cells were then washed to remove the inducers, further incubated for 84 h and the cell cycle profile was determined by flow cytometry (4 days after the induction). The fraction of cells in G1, G2 or polyploidy cells (>G2) is indicated in each case.
(TIF)

Figure S2 Interaction networks of genes regulated by p21 in K562 cells. A knowledge-based database (Ingenuity Pathways Analysis) was seeded with the genes regulated by p21 at 12 h of induction (Table S2). The two networks with the highest score are shown. The program processed 279 genes (137 up-regulated, 142 down-regulated). The ontogeny category of the networks is as indicated at the bottom. Genes in red were up-regulated and those in green were down-regulated. The meanings of node shape and lines are indicated at the bottom.
(TIF)

Figure S3 Comparison of the gene regulation mediated by p21 and p27 in K562 cells. p21 was induced in Kp21-4 cells and p27 was induced in Kp27-5 cells by 75 µM ZnSO_4_. After 3, 6 and 12 h of induction, total RNA was prepared and expression of the indicated genes was determined by RT-qPCR. The data for Kp21-4 cells are the same than in Figure 2. The values are means ±S.E.M. from two independent experiments and two determinations for each RNA
(TIF)

Figure S4 A. Gene expression regulation mediated by 12 h induction of p27 in K562 cells. The transcriptome of Kp27-5 cells (Kp27) treated for 12 h with ZnSO_4_ (to induce p27) were compared to that of cells with induced p21 (Kp21) and parental K562 treated for 12 h with ZnSO_4_. The heat map shows the hierarchical clustering with those genes with expression variation ≥2.3-fold between uninduced and p27-induced Kp27-5 cells after subtraction of the gene expression changes due to p21 in Kp21 cells(P<0.001). The heatmap shows 179 genes. B. Common genes regulated by both p21 and p27 in K562 cells. Kp21-4 and Kp27-5 cells were treated for 12 h with 75 µM ZnSO_4_ to induce p21 and p27 respectively. The heat map shows the hierarchical clustering with those genes with expression variation ≥2.3-fold between uninduced cells and Zn^2+^ –treated cells which are regulated in both cell lines(P<0.001). The heat map shows 90 genes. The genes related to cell cycle according with Gene Ontology are shown in red.
(TIF)

Table S1 Primers used in PCR reactions in this work. The forward primer is in the first line. All correspond to human genes except GFP (Green Fluorescent Protein from *Aequorea victoria*, encoded in the plasmid pEGFP-C1). An alternative common name of the gene is included for some genes.
(DOC)

Table S2 Genes regulated by p21 in K562 cells. The table list the genes showing an expression change in Kp21-4 cells treated with ZnSO_4_ (p21 inducer) for 12 h, after subtracting those genes changed by ZnSO_4_ in parental K562 cells (i.e., changed by ZnSO_4_) and in Kp27-5 cells (i.e., induced by p27). The 253 genes are included in the heat map of Fig. 1B. The table includes genes with ID and with a fold change ≥log_2_1.2 (≥2.3-fold) and with a signal difference ≥50 between both experimental conditions (as defined by dChip program and with Affymetrix U133 biochip data). Values are mean of fold changes (expressed as log_2_) of two independent experiments (P<0.001). For those genes represented by two or three Affymetrix probes, the fold change is the mean between the values of the probes. A negative fold change indicates down-regulation upon p21 induction.
(DOC)

Table S3 Genes regulated by p21 in primary keratinocytes. The table list the genes showing an expression change in human primary keratinocytes infected with adenovirus-p21 as described in Methods. RNA was analysed 24 h after infection. The 75 genes are included in the heat map of Fig. 7. The table includes genes with a fold change ≥log_2_1.2 (≥2.3-fold) and with a signal difference ≥50 between both experimental conditions (as defined by dChip program and with Affymetrix U133 biochip data). Values are mean of fold changes (expressed as log_2_) of two independent experiments (p<0.001). For those genes represented by two or three Affymetrix probes, the fold change is the mean between the values of the probes. A negative fold change indicates down-regulation upon p21 induction.
(DOC)
