# Supplementary figures and images for: p21 as a Transcriptional Co-Repressor of S-Phase and Mitotic Control Genes
Source: PLoS One. 2012 May 25;7(5):e37759. doi: 10.1371/journal.pone.0037759 (PMC3360621; doi:10.1371/journal.pone.0037759)

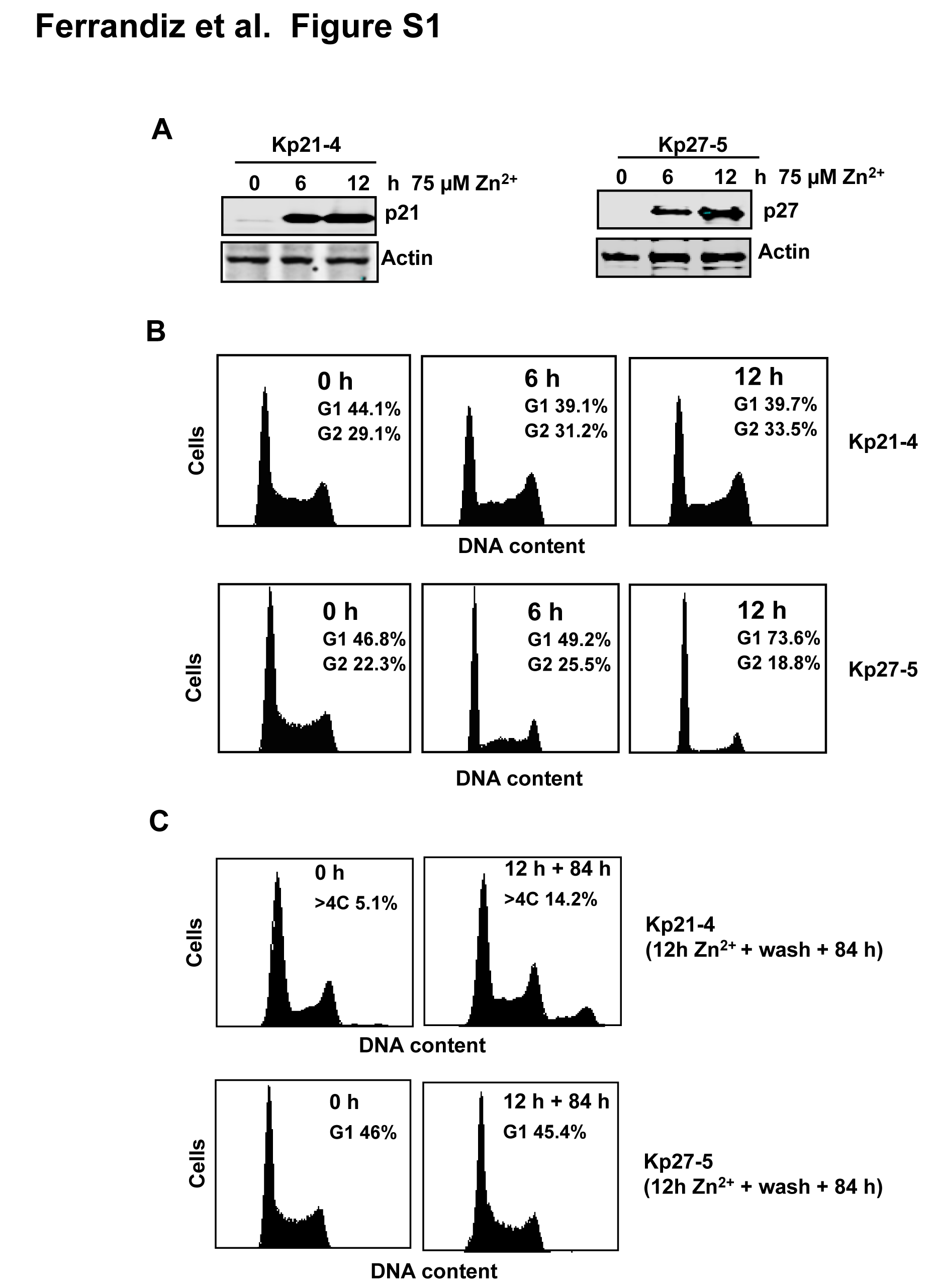

Supplement: Figure S1 — Cell cycle alterations mediated by p21 and p27 in K562 cells. A. Immunoblots showing the induction of p21 in Kp21-4 cells and p27 in Kp7-5 cells after 6 and 12 h of treatment with 75 µM ZnSO4. B. Absence of cell cycle profile alteration after short induction times of p21. Cell cycle profile of Kp21-4 and Kp27-5 cells upon induction of p21 and p27 with 75 µM ZnSO4 for 6 and 12 h. The cell cycle profile was determined by flow cytometry of propidium iodide-stained cells. C. p21 induces an irreversible accumulation of G2 and polyploid cells whereas p27 induces a reversible accumulation in G1. Kp21-4 and Kp27-5 were treated with ZnSO4 for 12 h. The cells were then washed to remove the inducers, further incubated for 84 h and the cell cycle profile was determined by flow cytometry (4 days after the induction). The fraction of cells in G1, G2 or polyploidy cells (>G2) is indicated in each case. (TIF) [file pone.0037759.s001.tif]

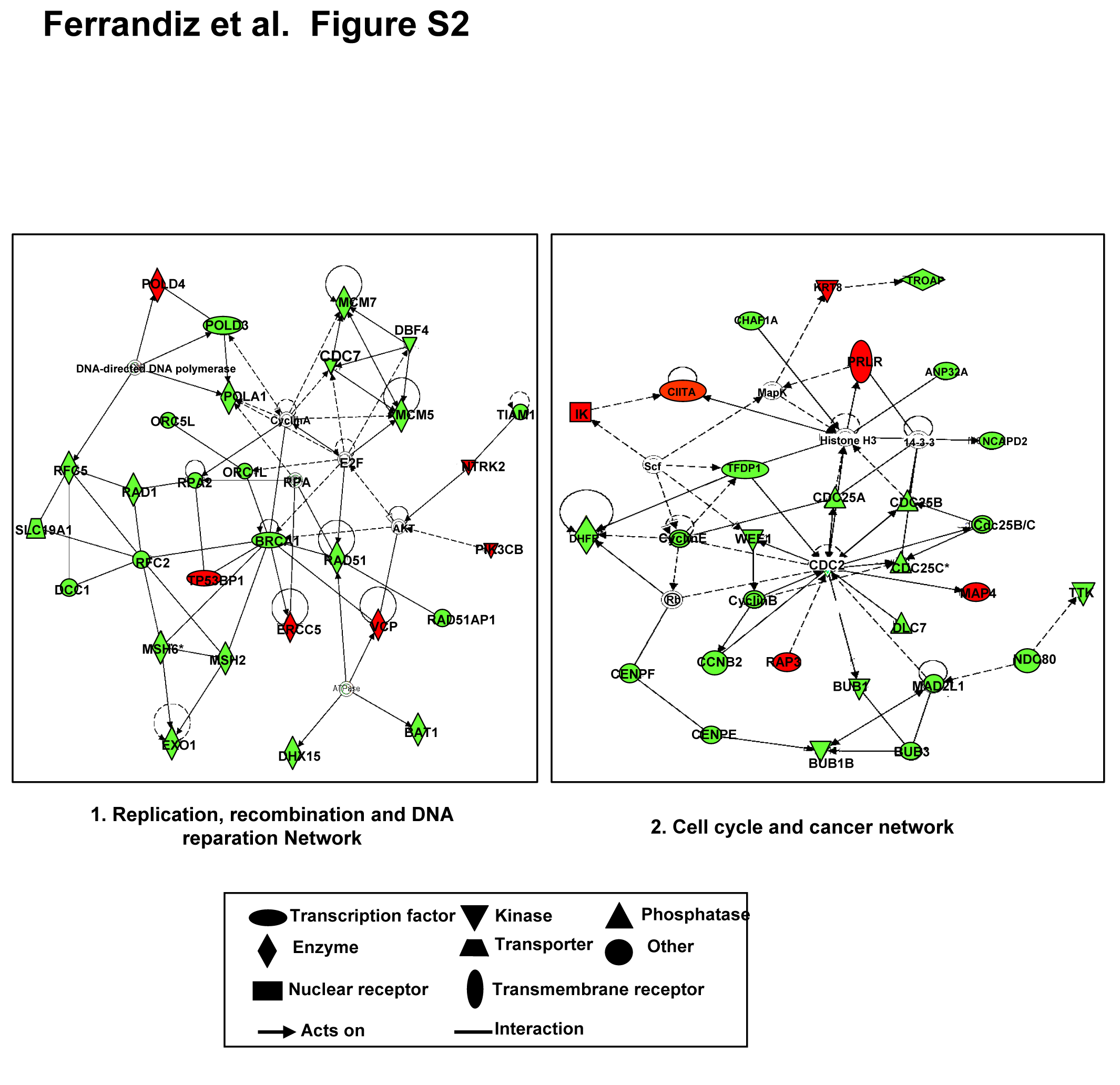

Supplement: Figure S2 — Interaction networks of genes regulated by p21 in K562 cells. A knowledge-based database (Ingenuity Pathways Analysis) was seeded with the genes regulated by p21 at 12 h of induction (Table S2). The two networks with the highest score are shown. The program processed 279 genes (137 up-regulated, 142 down-regulated). The ontogeny category of the networks is as indicated at the bottom. Genes in red were up-regulated and those in green were down-regulated. The meanings of node shape and lines are indicated at the bottom. (TIF) [file pone.0037759.s002.tif]

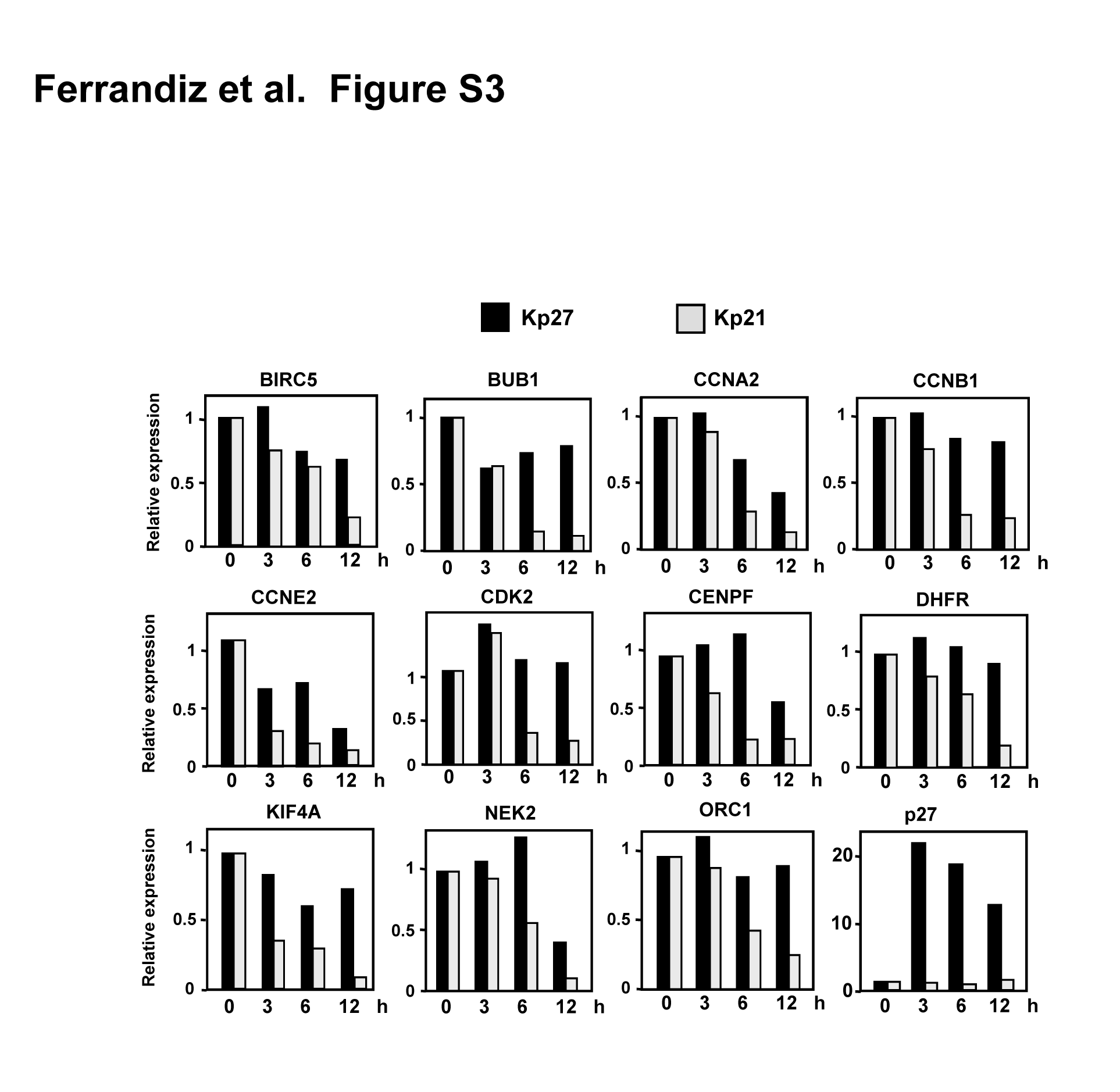

Supplement: Figure S3 — Comparison of the gene regulation mediated by p21 and p27 in K562 cells. p21 was induced in Kp21-4 cells and p27 was induced in Kp27-5 cells by 75 µM ZnSO4. After 3, 6 and 12 h of induction, total RNA was prepared and expression of the indicated genes was determined by RT-qPCR. The data for Kp21-4 cells are the same than in Figure 2. The values are means ±S.E.M. from two independent experiments and two determinations for each RNA (TIF) [file pone.0037759.s003.tif]

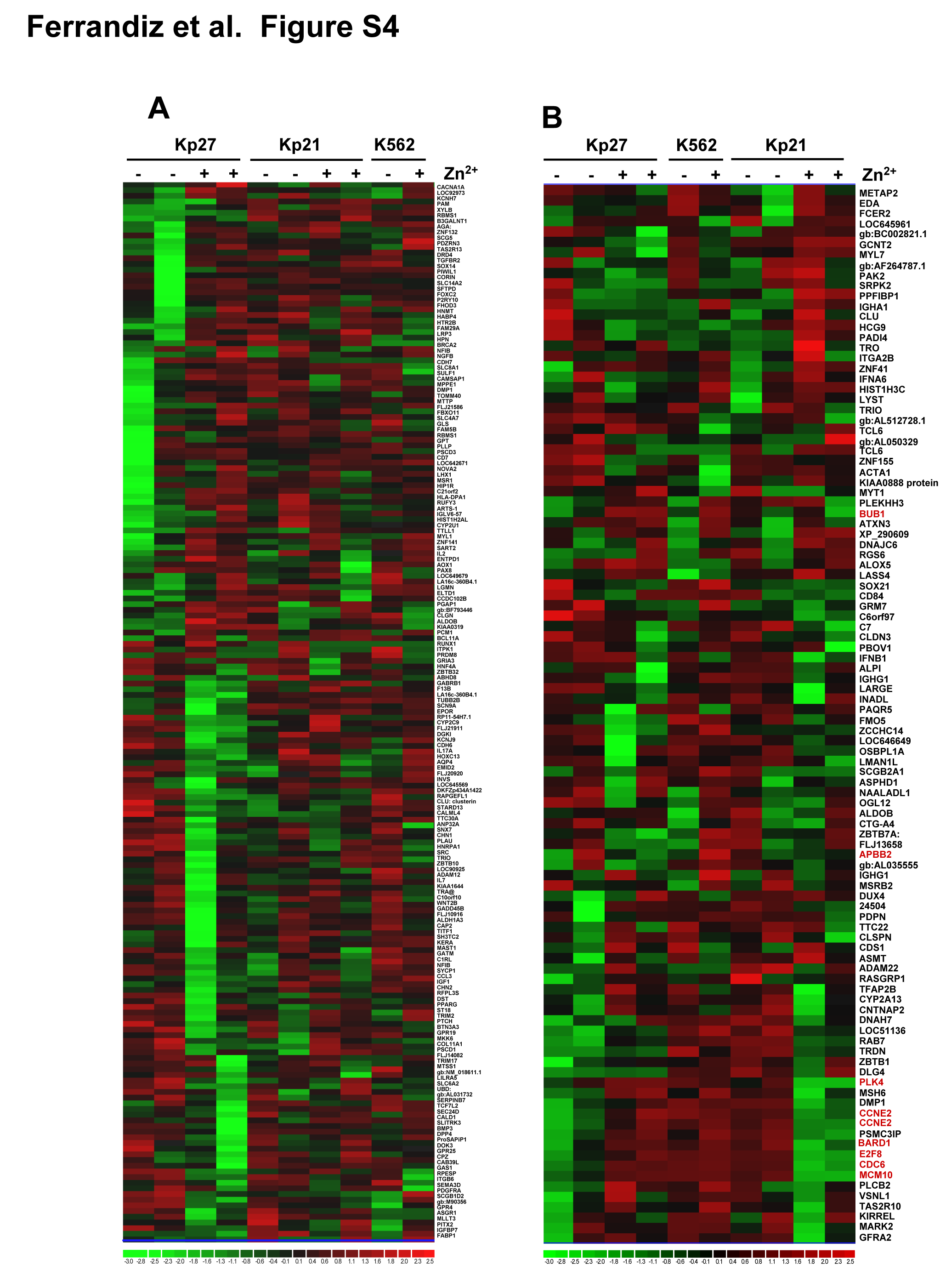

Supplement: Figure S4 — A. Gene expression regulation mediated by 12 h induction of p27 in K562 cells. The transcriptome of Kp27-5 cells (Kp27) treated for 12 h with ZnSO4 (to induce p27) were compared to that of cells with induced p21 (Kp21) and parental K562 treated for 12 h with ZnSO4. The heat map shows the hierarchical clustering with those genes with expression variation ≥2.3-fold between uninduced and p27-induced Kp27-5 cells after subtraction of the gene expression changes due to p21 in Kp21 cells(P<0.001). The heatmap shows 179 genes. B. Common genes regulated by both p21 and p27 in K562 cells. Kp21-4 and Kp27-5 cells were treated for 12 h with 75 µM ZnSO4 to induce p21 and p27 respectively. The heat map shows the hierarchical clustering with those genes with expression variation ≥2.3-fold between uninduced cells and Zn2+ –treated cells which are regulated in both cell lines(P<0.001). The heat map shows 90 genes. The genes related to cell cycle according with Gene Ontology are shown in red. (TIF) [file pone.0037759.s004.tif]
