# Supplementary material for: p21 as a Transcriptional Co-Repressor of S-Phase and Mitotic Control Genes
Source: PLoS One. 2012 May 25;7(5):e37759. doi: 10.1371/journal.pone.0037759 (PMC3360621; doi:10.1371/journal.pone.0037759)
Supplement: Table S1 — Primers used in PCR reactions in this work. The forward primer is in the first line. All correspond to human genes except GFP (Green Fluorescent Protein from Aequorea victoria, encoded in the plasmid pEGFP-C1). An alternative common name of the gene is included for some genes. (DOC) [file pone.0037759.s005.doc]

**Ferrandiz et al.**

**p21 as a transcriptional co-repressor of S-phase and mitotic control genes**

**Table S1**. **Primers used in PCR reactions in this work**. The forward primer is in the first line. All correspond to human genes except GFP (Green Fluorescent Protein from *Aequorea victoria*, encoded in the plasmid pEGFP-C1). An alternative common name of the gene is included for some genes.

| **Gene** | **Primer sequences (5’🡪3’)** | **Used for** | **Amplicon size** |
| --- | --- | --- | --- |
| BIRC5/Survivin | GTTGCGCTTTCCTTTCTGTC  TCCGCAGTTTCCTCAAATTC | RT-qPCR | 139 bp |
| BUB1B | CTCGTGGCAATACAGCTTCA  CAGGCTTTCTGGTGCTTAGG | RT-qPCR | 147 bp |
| BUB1B | GAGGAGCTACTGGCTCAAGG  AACCTGAACCGCAAACTAGAAG | ChIP | 179 bp |
| BUB3 | GTGGGACTTACGGAACATGG  CACTCGGCCTTCAATAGAGC | RT-qPCR | 127 bp |
| CCNA2/Cyclin A2 | TACCTGGACCCAGAAAACCA  CACTCACTGGCTTTTCATCTTCT | RT-qPCR | 114 bp |
| CCNA2/Cyclin A2 | GGCCCTAAATCCTACCTCTCC  CAGAGATGCAGCGAGCAG | ChIP | 243 bp |
| CCNB1/Cyclin B1 | TGTGGATGCAGAAGATGGAG  TGGCTCTCATGTTTCCAGTG | RT-qPCR | 146 bp |
| CCNB2/Cyclin B2 | TTCAACCCACCAAAACAACA  CTCAGGTGTGGGAGAAGGAC | RT-qPCR | 110 bp |
| CCNE1/Cyclin E1 | TTTTTGCAGGATCCAGATGA  TGCACGTTGAGTTTGGGTAA | RT-qPCR | 166 bp |
| CCNE2/Cyclin E2 | GGGAAACATTTTATCTTGCACA  CTGCAAGCACCATCAGTGAC | RT-qPCR | 178 bp |
| CCNE2/Cyclin E2 | CTACGCGCAGCAACTCCT  CTGTCCGGAGGTGTCAGTCT | ChIP | 130 bp |
| CDC25C | AGGTGCCCCTGGTTAGAATC  TCATCCACAAGAGAGGAAGGA | RT-qPCR | 155 bp |
| CDC25C | CAGAGTCTTCCCTGAGCAGAA  ATTGGCTGACGCAGCTTAGA | ChIP | 135 bp |
| CDK2 | TCCTCCACCGAGACCTTAAA  TACCACAGGGTCACCACCTC | RT-qPCR | 136 bp |
| CDK2 | CCCCAGGATTGGTGAAAATA  GGCCTTTCTATTGGTCAACG | ChIP | 230 bp |
| CDKN1A (p21) | GGAAGACCATGTGGACCTGT  GGCGTTTGGAGTGGTAGAAA | RT-qPCR | 146 bp |
| CDKN1B (p27) | CCGGCTAACTCTGAGGACAC  AGAAGAATCGTCGGTTGCAG | RT-qPCR | 120 bp |
| CENPF | AGGCGAGTCAGATCAAGGAG  GTTCTTGAGTTCTGCCTCCAG | RT-qPCR | 131 bp |
| DHFR | CAAGGAGCTCATTTTCTTTCCA  GTTTAAGATGGCCTGGGTGA | RT-qPCR | 148 bp |
| HIST4H4 | ATGTCTGGGCGAGGTAAAGG  GAGACCAGAAATGCGCTTGAC | RT-qPCR | 150 bp |
| KIF2C | TTCACAGTGCCAATGTAAGGA  GGGTTTATTGCAGCCACATC | RT-qPCR | 121 bp |
| KIF4A | AACAAGCGTCTCAAGGATGC  TTTCGTTTCCAAGCCAATTC-3´ | RT-qPCR | 121 bp |
| KIF4A | CAATTGGTTGGCGTCTCC  TAAGGTGTGCCCCCGTAAC | ChIP | 130 bp |
| MAD2L1 | CGGACTCACCTTGCTTGTAAC  TCCAGGACCTCACCACTTTC | RT-qPCR | 147 bp |
| MCM7 | GCCAAGTCTCAGCTCCTGTC  CCCACCCTCTAAGGTCAGTTC | RT-qPCR | 141 bp |
| NEK2/NIMA | AGCTTGGAGACTTTGGGCTA  AGCAGCCCAATGACCAGATA | RT-qPCR | 147 bp |
| ORC1 | CCAGCTGGTAGCCAGGAAAG  CCATTGAGTGGGATATGGTG | RT-qPCR | 143 bp |
| GFP | CGACGGCAACTACAAGACC  GCTTGTCGGCCATGATATAGA | RT-qPCR | 165 bp |
| PLK1 | CCTCCGGATCAAGAAGAATG  AGCAGCTCGTTAATGGTTGG | RT-qPCR | 120 bp |
| PLK1 | CCGTGTCAATCAGGTTTTCC  AGTCACTGCAGCACTCATGC | ChIP | 246 bp |
| rDNA | Agtcgggttgcttgggaatgc  CCCTTACGGTACTTGTTGACT | ChIP | 97 bp |
| RPS14 | TCACCGCCCTACACATCAAAC  TCCTGCGAGTGCTGTCAGAG | RT-qPCR | 161 bp |
| SKP2 | CATTTCCCAGTCAGCCGTAG  GCCCTTACTTCCTTCCCTTG | ChIP | 132 bp |
| AURKB | TCTCTAAGGATGGCCCAGAA  GCTCATGAGGACAAGTGCAG | RT-qPCR | 138 bp |
| AURKA | TTTCCATGATGCTACCAGAGTC  GGGCATTTGCCAATTCTG | RT-qPCR | 134 bp |
| WEE1 | CTTCAGCAATGGCACTGGTA  TTGCCATCTGTGCTTTCTTG | RT-qPCR | 141bp |
| WEE1 | TGGAAGAAACGTGTAAGTGCAT  TTACATTTGTTCGCCAGCAC | ChIP | 148 bp |
