# Supplementary material for: p21 as a Transcriptional Co-Repressor of S-Phase and Mitotic Control Genes
Source: PLoS One. 2012 May 25;7(5):e37759. doi: 10.1371/journal.pone.0037759 (PMC3360621; doi:10.1371/journal.pone.0037759)
Supplement: Table S2 — Genes regulated by p21 in K562 cells. The table list the genes showing an expression change in Kp21-4 cells treated with ZnSO4 (p21 inducer) for 12 h, after subtracting those genes changed by ZnSO4 in parental K562 cells (i.e., changed by ZnSO4) and in Kp27-5 cells (i.e., induced by p27). The 253 genes are included in the heat map of Fig. 1B. The table includes genes with ID and with a fold change ≥log21.2 (≥2.3-fold) and with a signal difference ≥50 between both experimental conditions (as defined by dChip program and with Affymetrix U133 biochip data). Values are mean of fold changes (expressed as log2) of two independent experiments (P<0.001). For those genes represented by two or three Affymetrix probes, the fold change is the mean between the values of the probes. A negative fold change indicates down-regulation upon p21 induction. (DOC) [file pone.0037759.s006.doc]

**Ferrandiz et al.**

**p21 as a transcriptional co-repressor of S-phase and mitotic control genes**

**Table S2. Genes regulated by p21 in K562 cells**. The table list the genes showing an expression change in Kp21-4 cells treated with ZnSO_4_ (p21 inducer) for 12 h, after subtracting those genes changed by ZnSO_4_ in parental K562 cells (i.e., changed by ZnSO_4_) and in Kp27-5 cells (i.e., induced by p27). The 253 genes are included in the heat map of Fig. 1B. The table includes genes with ID and with a fold change ≥log_2_1.2 (≥2.3-fold) and with a signal difference ≥50 between both experimental conditions (as defined by dChip program and with Affymetrix U133 biochip data). Values are mean of fold changes (expressed as log_2_) of two independent experiments (P<0.001). For those genes represented by two or three Affymetrix probes, the fold change is the mean between the values of the probes. A negative fold change indicates down-regulation upon p21 induction.

| **Symbol** | **Gene Description** | **Gene ID** | **Fold change (log2)** |
| --- | --- | --- | --- |
| SLC16A4 | solute carrier family 16 (monocarboxylic acid transporters), member 4 | 9122 | 6.47 |
| CRIP2 | Hypothetical protein MGC27165 | 283650 | 5.02 |
| PHF20L1 | PHD finger protein 20-like 1 | 51105 | 3.78 |
| RGS5 | regulator of G-protein signalling 5 | 8490 | 3.21 |
| KCTD14 | potassium channel tetramerisation domain containing 14 | 65987 | 3.17 |
| EHBP1 | EH domain binding protein 1 | 23301 | 3.13 |
| NTRK2 | neurotrophic tyrosine kinase, receptor, type 2 | 4915 | 3.04 |
| CRIP2 | cysteine-rich protein 2 | 1397 | 2.73 |
| ACOX | acyl-Coenzyme A oxidase 1, palmitoyl | 51 | 2.69 |
| CD3G | CD3G antigen, gamma polypeptide (TiT3 complex) | 917 | 2.38 |
| GOLGA2 | golgi autoantigen, golgin subfamily a, 2 | 2801 | 2.34 |
| AKR1C4 | aldo-keto reductase family 1, member C4 | 1109 | 2.34 |
| HCG9 | HLA complex group 9 | 10255 | 2.29 |
| SLC22A18 | solute carrier family 22 (organic cation transporter), member 18 | 5002 | 2.27 |
| WIPF1 | Wiskott-Aldrich syndrome protein interacting protein | 7456 | 2.26 |
| OSBPL1A | oxysterol binding protein-like 1A | 114876 | 2.26 |
| LGALS8 | lectin, galactoside-binding, soluble, 8 (galectin 8) | 3964 | 2.20 |
| TGFB2 | transforming growth factor, beta 2 | 7042 | 2.19 |
| EHD1 | EH-domain containing 1 | 10938 | 2.18 |
| TF | transferrin | 7018 | 2.14 |
| TFE3 | transcription factor binding to IGHM enhancer 3 | 7030 | 2.11 |
| CAPN3 | calpain 3, (p94) | 825 | 2.10 |
| CEP63 | centrosome protein Cep63 | 80254 | 2.05 |
| SLCO2B1 | solute carrier organic anion transporter family, member 2B1 | 11309 | 2.04 |
| LRP12 | low density lipoprotein-related protein 12 | 29967 | 2.03 |
| ABHD2 | abhydrolase domain containing 2 | 11057 | 2.03 |
| ZNF324 | zinc finger protein 324 | 25799 | 2.01 |
| LRP12 | low density lipoprotein-related protein 12 | 29967 | 2.00 |
| TANK | TRAF family member-associated NFKB activator | 10010 | 1.99 |
| HPGD | hydroxyprostaglandin dehydrogenase 15-(NAD) | 3248 | 1.98 |
| SHC1 | SHC (Src homology 2 domain containing) transforming protein 1 | 6464 | 1.98 |
| CIITA | MHC class II transactivator | 4261 | 1.97 |
| MLLT11 | ALL1-fused gene from chromosome 1q | 10962 | 1.97 |
| KIAA1609 | KIAA1609 protein | 57707 | 1.95 |
| GOLGA2 | golgi autoantigen, golgin subfamily a, 2 | 2801 | 1.95 |
| BRWD1 | WD repeat domain 9 | 54014 | 1.91 |
| TP53BP1 | tumor protein p53 binding protein, 1 | 7158 | 1.92 |
| ZER1 | chromosome 9 open reading frame 60 | 10444 | 1.89 |
| ABHD2 | abhydrolase domain containing 2 | 11057 | 1.88 |
| MAT1A | methionine adenosyltransferase I, alpha | 4143 | 1.87 |
| MED25 | mediator of RNA polymerase II, subunit 25 homolog (yeast) | 81857 | 1.87 |
| KIAA0404 | KIAA0404 protein | 23130 | 1.86 |
| CCHCR1 | coiled-coil alpha-helical rod protein 1 | 54535 | 1.85 |
| HDAC5 | histone deacetylase 5 | 10014 | 1.85 |
| MEIS1 | Meis1, myeloid ecotropic viral integration site 1 homolog (mouse) | 4211 | 1.83 |
| LOC92482 | hypothetical protein LOC92482 | 92482 | 1.82 |
| AKR1C2 | aldo-keto reductase family 1, member C2 | 1646 | 1.81 |
| LAMB3 | laminin, beta 3 | 3914 | 1.81 |
| PRLR | prolactin receptor | 5618 | 1.80 |
| RRBP1 | Ribosome binding protein 1 homolog 180kDa (dog) | 6238 | 1.78 |
| CRAT | carnitine acetyltransferase | 1384 | 1.78 |
| WDR37 | WD repeat domain 37 | 22884 | 1.78 |
| STX12 | syntaxin 12 | 23673 | 1.78 |
| SCAMP1 | secretory carrier membrane protein 1 | 9522 | 1.76 |
| ACADSB | acyl-Coenzyme A dehydrogenase, short/branched chain | 36 | 1.74 |
| MLN | motilin | 4295 | 1.74 |
| SALL2 | sal-like 2 (Drosophila) | 6297 | 1.74 |
| DPP3 | dipeptidylpeptidase 3 | 10072 | 1.74 |
| TMEM134 | transmembrane protein 134 | 80194 | 1.73 |
| PLCL1 | phospholipase C-like 1 | 5334 | 1.72 |
| RBM8A | RNA binding motif protein 8A | 9939 | 1.72 |
| RCBTB2 | chromosome condensation 1-like | 1102 | 1.71 |
| CDH19 | cadherin 19, type 2 | 28513 | 1.71 |
| CSF1 | colony stimulating factor 1 (macrophage) | 1435 | 1.71 |
| ARHGEF6 | Rac/Cdc42 guanine nucleotide exchange factor (GEF) 6 | 9459 | 1.71 |
| ALDH1A1 | aldehyde dehydrogenase 1 family, member A1 | 216 | 1.71 |
| GTF3C1 | general transcription factor IIIC, polypeptide 1, alpha 220kDa | 2975 | 1.70 |
| ERCC5 | excision repair cross-complementing repair deficiency, complementation group 5 | 2073 | 1.70 |
| ZMIZ1 | retinoic acid induced 17 | 57178 | 1.68 |
| LOC282997 | hypothetical protein LOC282997 | 282997 | 1.68 |
| CPNE3 | copine III | 8895 | 1.67 |
| KLHDC2 | kelch domain containing 2 | 23588 | 1.67 |
| EIF1AY | eukaryotic translation initiation factor 1A, Y-linked | 9086 | 1.66 |
| SLC35A3 | solute carrier family 35 (UDP-N-acetylglucosamine) transporter), A3 | 23443 | 1.66 |
| MAPRE3 | microtubule-associated protein, RP/EB family, member 3 | 22924 | 1.65 |
| PPP2R5B | protein phosphatase 2, regulatory subunit B (B56), beta isoform | 5526 | 1.65 |
| CYB5R1 | NAD(P)H:quinone oxidoreductase type 3, polypeptide A2 | 51706 | 1.64 |
| GLRX | glutaredoxin (thioltransferase) | 2745 | 1.64 |
| GYS1 | glycogen synthase 1 (muscle) | 2997 | 1.63 |
| GABRE | gamma-aminobutyric acid (GABA) A receptor, epsilon | 2564 | 1.63 |
| LGALS8 | lectin, galactoside-binding, soluble, 8 (galectin 8) | 3964 | 1.63 |
| COPB2 | coatomer protein complex, subunit beta 2 (beta prime) | 9276 | 1.62 |
| LOH11CR2A | loss of heterozygosity, 11, chromosomal region 2, gene A | 4013 | 1.62 |
| UBR2 | ubiquitin protein ligase E3 component n-recognin 2 | 23304 | 1.62 |
| MBTPS1 | membrane-bound transcription factor protease, site 1 | 8720 | 1.61 |
| CYBA | cytochrome b-245, alpha polypeptide | 1535 | 1.60 |
| CDK10 | cyclin-dependent kinase (CDC2-like) 10 | 8558 | 1.60 |
| STX4 | syntaxin 4A (placental) | 6810 | 1.60 |
| HARSL | histidyl-tRNA synthetase-like | 23438 | 1.60 |
| SOX12 | SRY (sex determining region Y)-box 12 | 6666 | 1.59 |
| LIG4 | ligase IV, DNA, ATP-dependent | 3981 | 1.59 |
| ME1 | malic enzyme 1, NADP(+)-dependent, cytosolic | 4199 | 1.58 |
| CPNE3 | copine III | 8895 | 1.57 |
| ATP2C1 | ATPase, Ca++ transporting, type 2C, member 1 | 27032 | 1.57 |
| CNOT2 | CCR4-NOT transcription complex, subunit 2 | 4848 | 1.57 |
| BC058818 | Chloride channel 3 | 1182 | 1.56 |
| VPS16 | vacuolar protein sorting 16 (yeast) | 64601 | 1.56 |
| EML3 | echinoderm microtubule associated protein like 3 | 256364 | 1.56 |
| MAN2C1 | mannosidase, alpha, class 2C, member 1 | 4123 | 1.55 |
| TRIP4 | thyroid hormone receptor interactor 4 | 9325 | 1.55 |
| NUMB | numb homolog (Drosophila) | 8650 | 1.55 |
| JMJD2B | jumonji domain containing 2B | 23030 | 1.55 |
| SWAP70 | SWAP-70 protein | 23075 | 1.54 |
| ZNF32 | zinc finger protein 32 (KOX 30) | 7580 | 1.54 |
| TRPM4 | transient receptor potential cation channel, subfamily M, member 4 | 54795 | 1.54 |
| CTR9 | SH2 domain binding protein 1 (tetratricopeptide repeat containing) | 9646 | 1.53 |
| STXBP3 | syntaxin binding protein 3 | 6814 | 1.53 |
| RABL2B | RAB, member of RAS oncogene family-like 2B | 11158 | 1.52 |
| GSN | gelsolin (amyloidosis, Finnish type) | 2934 | 1.51 |
| SPTLC2 | serine palmitoyltransferase, long chain base subunit 2 | 9517 | 1.51 |
| HS2ST1 | heparan sulfate 2-O-sulfotransferase 1 | 9653 | 1.51 |
| AHNAK | AHNAK nucleoprotein (desmoyokin) | 79026 | 1.51 |
| IK | IK cytokine, down-regulator of HLA II | 3550 | 1.50 |
| AK074259 | Pleckstrin homology domain containing, family B member 2 | 55041 | 1.50 |
| MEF2C | MADS box transcription enhancer factor 2, polypeptide C | 4208 | 1.50 |
| NLK | nemo like kinase | 51701 | 1.50 |
| IFT52 | chromosome 20 open reading frame 9 | 51098 | 1.50 |
| PEX19 | peroxisomal biogenesis factor 19 | 5824 | 1.49 |
| ZFP95 | zinc finger protein 95 homolog (mouse) | 23660 | 1.49 |
| IFNGR1 | interferon gamma receptor 1 | 3459 | 1.49 |
| FNBP1 | formin binding protein 1 | 23048 | 1.49 |
| MICAL2 | microtubule associated monoxygenase, calponin and LIM domain 2 | 9645 | 1.49 |
| IGHD | immunoglobulin heavy constant delta | 3495 | 1.49 |
| GSTM2 | glutathione S-transferase M2 (muscle) | 2946 | 1.48 |
| DNAJB6 | DnaJ (Hsp40) homolog, subfamily B, member 6 | 10049 | 1.48 |
| NIPSNAP3B | nipsnap homolog 3B (C. elegans) | 55335 | 1.48 |
| DCTN2 | dynactin 2 (p50) | 10540 | 1.47 |
| ACBD3 | acyl-Coenzyme A binding domain containing 3 | 64746 | 1.47 |
| SLC25A40 | mitochondrial carrier family protein | 55972 | 1.47 |
| KDELR3 | KDEL (Lys-Asp-Glu-Leu) endoplasmic reticulum protein retention receptor 3 | 11015 | 1.47 |
| ZNF289 | zinc finger protein 289, ID1 regulated | 84364 | 1.47 |
| SERPINB1 | serine proteinase inhibitor, clade B (ovalbumin), member 1 | 1992 | 1.47 |
| PIK3CB | phosphoinositide-3-kinase, catalytic, beta polypeptide | 5291 | 1.47 |
| ARL6IP5 | ADP-ribosylation-like factor 6 interacting protein 5 | 10550 | 1.46 |
| KIAA1033 | KIAA1033 | 23325 | 1.46 |
| WDR26 | WD repeat domain 26 | 80232 | 1.46 |
| MECR | nuclear receptor binding factor 1 | 51102 | 1.46 |
| IPPK | chromosome 9 open reading frame 12 | 64768 | 1.46 |
| RBKS | ribokinase | 64080 | 1.45 |
| TOB1 | transducer of ERBB2, 1 | 10140 | 1.45 |
| BCL2L1 | BCL2-like 1 | 598 | 1.45 |
| PRMT3 | HMT1 hnRNP methyltransferase-like 3 (S. cerevisiae) | 10196 | 1.45 |
| PLSCR3 | phospholipid scramblase 3 | 57048 | 1.44 |
| POLD4 | polymerase (DNA-directed), delta 4 | 57804 | 1.44 |
| CASP9 | caspase 9, apoptosis-related cysteine protease | 842 | 1.44 |
| CCNG1 | cyclin G1 | 900 | 1.44 |
| PSCD2 | pleckstrin homology, Sec7 and coiled-coil domains 2 (cytohesin-2) | 9266 | 1.44 |
| RHOQ | ras homolog gene family, member Q | 23433 | 1.44 |
| TAOK3 | TAO kinase 3 | 51347 | 1.44 |
| RAB11A | RAB11A, member RAS oncogene family | 8766 | 1.43 |
| SPATA2 | spermatogenesis associated 2 | 9825 | 1.43 |
| CLPX | ClpX caseinolytic protease X homolog (E. coli) | 10845 | 1.43 |
| DYNC1H1 | dynein, cytoplasmic, heavy polypeptide 1 | 1778 | 1.42 |
| TRAF3IP2 | TRAF3 interacting protein 2 | 10758 | 1.42 |
| SERINC3 | serine incorporator 3 | 10955 | 1.41 |
| RELA | v-rel reticuloendotheliosis viral oncogene homolog A, nuclear factor of kappa light polypeptide gene enhancer in B-cells 3 | 5970 | 1.41 |
| SLC4A1AP | solute carrier family 4 (anion exchanger), member 1 adaptor protein | 22950 | 1.40 |
| ACTR2 | ARP2 actin-related protein 2 homolog (yeast) | 10097 | 1.39 |
| MYB | v-myb myeloblastosis viral oncogene homolog (avian) | 4602 | 1.39 |
| VCP | valosin-containing protein | 7415 | 1.39 |
| BECN1 | beclin 1 (coiled-coil, myosin-like BCL2 interacting protein) | 8678 | 1.39 |
| C20orf44 | chromosome 20 open reading frame 44 | 55245 | 1.39 |
| VPS13C | vacuolar protein sorting 13C (yeast) | 54832 | 1.39 |
| SERINC1 | tumor differentially expressed 2 | 57515 | 1.38 |
| C9orf6 | hypothetical protein FLJ20457 | 54942 | 1.38 |
| ACO2 | aconitase 2, mitochondrial | 50 | 1.37 |
| PARP4 | poly (ADP-ribose) polymerase family, member 4 | 143 | 1.37 |
| MFSD1 | major facilitator superfamily domain containing 1 | 64747 | 1.37 |
| CTSB | cathepsin B | 1508 | 1.36 |
| AHCYL1 | S-adenosylhomocysteine hydrolase-like 1 | 10768 | 1.35 |
| FLJ20254 | Hypothetical protein FLJ20254 | 54867 | 1.35 |
| KRT8 | keratin 8 | 3856 | 1.33 |
| PRKCB1 | protein kinase C, beta 1 | 5579 | 1.33 |
| CDK5RAP3 | CDK5 regulatory subunit associated protein 3 | 80279 | 1.32 |
| KIAA0196 | KIAA0196 gene product | 9897 | 1.31 |
| MAP4 | microtubule-associated protein 4 | 4134 | 1.30 |
| UMPS | uridine monophosphate synthetase (orotate phosphoribosyl transferase and orotidine-5'-decarboxylase) | 7372 | -1.31 |
| ANP32A | Acidic (leucine-rich) nuclear phosphoprotein 32 family, member A | 8125 | -1.35 |
| TUBB2C | tubulin, beta, 2 | 10383 | -1.36 |
| UBE2N | ubiquitin-conjugating enzyme E2N (UBC13 homolog, yeast) | 7334 | -1.37 |
| COCH | coagulation factor C homolog, cochlin (Limulus polyphemus) | 1690 | -1.37 |
| FUSIP1 | FUS interacting protein (serine-arginine rich) 1 | 10772 | -1.38 |
| C20orf67 | Cluster Incl. AI743331:wg91f07. |  | -1.39 |
| PHF10 | PHD finger protein 10 | 55274 | -1.40 |
| DDX398 | DEAD (Asp-Glu-Ala-Asp) box polypeptide 39B | 7919 | -1.41 |
| PPM1G | protein phosphatase 1G (formerly 2C), magnesium-dependent, gamma isoform | 5496 | -1.41 |
| C7orf24 | chromosome 7 open reading frame 24 | 79017 | -1.43 |
| PXMP2 | peroxisomal membrane protein 2, 22kDa | 5827 | -1.43 |
| SMC1A | SMC1 structural maintenance of chromosomes 1-like 1 (yeast) | 8243 | -1.44 |
| SLC19A1 | solute carrier family 19 (folate transporter), member 1 | 6573 | -1.44 |
| PARP2 | poly (ADP-ribose) polymerase family, member 2 | 10038 | -1.44 |
| NR2F2 | nuclear receptor subfamily 2, group F, member 2 | 7026 | -1.45 |
| KLHL23 | kelch-like 23 (Drosophila) | 151230 | -1.45 |
| ACTL6A | actin-like 6A | 86 | -1.46 |
| CAD | carbamoyl-phosphate synthetase 2, aspartate transcarbamylase, and dihydroorotase | 790 | -1.46 |
| PNN | pinin, desmosome associated protein | 5411 | -1.46 |
| CHORDC1 | Cysteine- histidine-rich domain (CHORD) containing, zinc binding protein 1 | 26973 | -1.46 |
| KPNA2 | karyopherin alpha 2 (RAG cohort 1, importin alpha 1) | 3838 | -1.47 |
| NUDT15 | nudix (nucleoside diphosphate linked moiety X)-type motif 15 | 55270 | -1.47 |
| SPHK1 | sphingosine kinase 1 | 8877 | -1.49 |
| CDC25B | cell division cycle 25B | 994 | -1.50 |
| UMPS | uridine monophosphate synthetase | 7372 | -1.51 |
| CTPS | CTP synthase | 1503 | -1.52 |
| RAD1 | RAD1 homolog (S. pombe) | 5810 | -1.52 |
| DHX15 | DEAH (Asp-Glu-Ala-His) box polypeptide 15 | 1665 | -1.53 |
| RPL39L | ribosomal protein L39-like | 116832 | -1.53 |
| TMEM97 | hypothetical protein MAC30 | 27346 | -1.53 |
| LOC221362 | hypothetical protein LOC221362 | 221362 | -1.53 |
| GCH1 | GTP cyclohydrolase 1 (dopa-responsive dystonia) | 2643 | -1.54 |
| TLR2 | toll-like receptor 2 | 7097 | -1.55 |
| ESPL1 | extra spindle poles like 1 (S. cerevisiae) | 9700 | -1.56 |
| RPA2 | replication protein A2, 32kDa | 6118 | -1.56 |
| BUB3 | BUB3 budding uninhibited by benzimidazoles 3 homolog (yeast) | 9184 | -1.57 |
| HNRPAB | heterogeneous nuclear ribonucleoprotein A/B | 3182 | -1.58 |
| THOC1 | THO complex 1 | 9984 | -1.58 |
| CTSC | cathepsin C | 1075 | -1.59 |
| DHFR | dihydrofolate reductase | 1719 | -1.59 |
| HIVEP1 | human immunodeficiency virus type I enhancer binding protein 1 | 3096 | -1.59 |
| POLD3 | polymerase (DNA-directed), delta 3, accessory subunit | 10714 | -1.59 |
| DSN1 | chromosome 20 open reading frame 172 | 79980 | -1.59 |
| VRK1 | vaccinia related kinase 1 | 7443 | -1.60 |
| CKS2 | CDC28 protein kinase regulatory subunit 2 | 1164 | -1.60 |
| DBF4 | activator of S phase kinase | 10926 | -1.60 |
| DUT | dUTP pyrophosphatase | 1854 | -1.60 |
| MARCKSL1 | MARCKS-like 1 | 65108 | -1.61 |
| DHFR | dihydrofolate reductase | 1719 | -1.61 |
| TEAD4 | TEA domain family member 4 | 7004 | -1.61 |
| NCAPH2 | hypothetical protein 384D8_6 | 29781 | -1.61 |
| DBNDD2 | chromosome 20 open reading frame 35 | 55861 | -1.62 |
| EXO1 | exonuclease 1 | 9156 | -1.65 |
| KNTC1 | kinetochore associated 1 | 9735 | -1.65 |
| UCHL5IP | three prime repair exonuclease 2 | 11219 | -1.65 |
| CDCA4 | cell division cycle associated 4 | 55038 | -1.65 |
| BUB3 | BUB3 budding uninhibited by benzimidazoles 3 homolog (yeast) | 9184 | -1.67 |
| WHSC1 | Wolf-Hirschhorn syndrome candidate 1 | 7468 | -1.67 |
| MAFB | v-maf musculoaponeurotic fibrosarcoma oncogene homolog B (avian) | 9935 | -1.67 |
| DNMT1 | DNA (cytosine-5-)-methyltransferase 1 | 1786 | -1.69 |
| RBBP8 | retinoblastoma binding protein 8 | 5932 | -1.69 |
| CIT | citron (rho-interacting, serine/threonine kinase 21) | 11113 | -1.69 |
| MCM7 | MCM7 minichromosome maintenance deficient 7 (S. cerevisiae) | 4176 | -1.71 |
| CCDC99 | hypothetical protein FLJ20364 | 54908 | -1.71 |
| IMPA2 | inositol(myo)-1(or 4)-monophosphatase 2 | 3613 | -1.73 |
| SLC29A1 | solute carrier family 29 (nucleoside transporters), member 1 | 2030 | -1.75 |
| BTG3 | BTG family, member 3 | 10950 | -1.75 |
| NR2F2 | nuclear receptor subfamily 2, group F, member 2 | 7026 | -1.75 |
| SLC29A1 | solute carrier family 29 (nucleoside transporters), member 1 | 2030 | -1.76 |
| NCAPD2 | chromosome condensation-related SMC-associated protein 1 | 9918 | -1.78 |
| PMAIP1 | phorbol-12-myristate-13-acetate-induced protein 1 | 5366 | -1.79 |
| AGR2 | anterior gradient 2 homolog (Xenopus laevis) | 10551 | -1.80 |
| KIAA0101 | KIAA0101 /p15(PAF) | 9768 | -1.82 |
| NUP210 | nucleoporin 210kDa | 23225 | -1.83 |
| PLSCR1 | phospholipid scramblase 1 | 5359 | -1.84 |
| MSH6 | mutS homolog 6 (E. coli) | 2956 | -1.84 |
| DHRS2 | dehydrogenase/reductase (SDR family) member 2 | 10202 | -1.84 |
| PTMA | prothymosin, alpha (gene sequence 28) | 440085 | -1.84 |
| MSH6 | mutS homolog 6 (E. coli) | 2956 | -1.84 |
| CKAP2 | cytoskeleton associated protein 2 | 26586 | -1.84 |
| TCOF1 | Treacher Collins-Franceschetti syndrome 1 | 6949 | -1.85 |
| LAMA4 | laminin, alpha 4 | 3910 | -1.85 |
| TMEM97 | hypothetical protein MAC30 | 27346 | -1.85 |
| MSH2 | mutS homolog 2, colon cancer, nonpolyposis type 1 (E. coli) | 4436 | -1.86 |
| BTG3 | BTG family, member 3 | 10950 | -1.86 |
| NCAPD3 | non-SMC condensin II complex, subunit D3 | 23310 | -1.87 |
| CD200 | CD200 antigen | 4345 | -1.90 |
| FAM111A | family with sequence similarity 111, member | 63901 | -1.90 |
| SMC4 | SMC structural maintenance of chromosomes 4 | 10051 | -1.91 |
| UBE2S | ubiquitin-conjugating enzyme E2S | 27338 | -1.91 |
| POLA1 | polymerase (DNA directed), alpha | 5422 | -1.92 |
| BRCA1 | breast cancer 1, early onset | 672 | -1.94 |
| DTYMK | deoxythymidylate kinase (thymidylate kinase) | 1841 | -1.95 |
| SPAG5 | sperm associated antigen 5 | 10615 | -1.99 |
| TROAP | trophinin associated protein (tastin) | 10024 | -2.01 |
| SLC43A3 | solute carrier family 43, member 3 | 29015 | -2.01 |
| TRAC | T cell receptor alpha locus | 28755 | -2.02 |
| MCM6 | MCM6 minichromosome maintenance deficient 6 | 4175 | -2.03 |
| MAD2L1 | MAD2 mitotic arrest deficient-like 1 (yeast) | 4085 | -2.03 |
| RFC5 | replication factor C (activator 1) 5, 36.5kDa | 5985 | -2.04 |
| TRAIP | TRAF interacting protein | 10293 | -2.04 |
| AURKA | Aurore kinase A/serine/threonine kinase 6 | 6790 | -2.04 |
| RFC2 | replication factor C (activator 1) 2, 40kDa | 5982 | -2.08 |
| NASP | nuclear autoantigenic sperm protein (histone-binding) | 4678 | -2.11 |
| GINS3 | hypothetical protein FLJ13912 | 64785 | -2.11 |
| TFDP1 | transcription factor Dp-1 | 7027 | -2.17 |
| KIAA1794 | hypothetical protein FLJ10719 | 55215 | -2.17 |
| WDHD1 | WD repeat and HMG-box DNA binding protein 1 | 11169 | -2.18 |
| HIST1H2BG | histone 1, H2bg | 8339 | -2.19 |
| CENPN | uncharacterized bone marrow protein BM039 | 55839 | -2.19 |
| CCNF | cyclin F | 899 | -2.22 |
| DONSON | downstream neighbor of SON | 29980 | -2.22 |
| DEK | DEK oncogene (DNA binding) | 7913 | -2.24 |
| CCNB2 | cyclin B2 | 9133 | -2.24 |
| CDC25C | cell division cycle 25C | 995 | -2.26 |
| CENPM | chromosome 22 open reading frame 18 | 79019 | -2.26 |
| CENPQ | chromosome 6 open reading frame 139 | 55166 | -2.28 |
| CHAF1A | chromatin assembly factor 1, subunit A (p150) | 10036 | -2.29 |
| KIAA0286 | KIAA0286 protein | 23306 | -2.30 |
| CDC25C | cell division cycle 25C | 995 | -2.31 |
| ORC1 | origin recognition complex, subunit 1-like (yeast) | 4998 | -2.33 |
| RACGAP1 | Rac GTPase activating protein 1 | 29127 | -2.35 |
| HMGB3 | high-mobility group box 3 | 3149 | -2.41 |
| B3GALNT1 | UDP-Gal:betaGlcNAc beta 1,3-galactosyltransferase, polypeptide 3 | 8706 | -2.46 |
| WEE1 | WEE1 homolog (S. pombe) | 7465 | -2.47 |
| CDT1 | DNA replication factor | 81620 | -2.48 |
| FHL1 | four and a half LIM domains 1 | 2273 | -2.48 |
| NCAPG2 | more than blood homolog | 54892 | -2.49 |
| CYP2C18 | cytochrome P450, family 2, subfamily C, polypeptide 18 | 1562 | -2.54 |
| LOC146909 | hypothetical protein LOC146909 | 146909 | -2.54 |
| AK124872 | Similar to BMP2 inducible kinase | 388957 | -2.60 |
| CDC7 | CDC7 cell division cycle 7 (S. cerevisiae) | 8317 | -2.61 |
| CENPF | centromere protein F, 350/400ka (mitosin) | 1063 | -2.63 |
| CCNF | cyclin F | 899 | -2.64 |
| WDHD1 | WD repeat and HMG-box DNA binding protein 1 | 11169 | -2.68 |
| HJURP | Holliday junction recognition protein | 55355 | -2.71 |
| BUB1 | BUB1 budding uninhibited by benzimidazoles 1 homolog (yeast) | 699 | -2.72 |
| MKI67 | antigen identified by monoclonal antibody Ki-67 | 4288 | -2.77 |
| HMMR | hyaluronan-mediated motility receptor (RHAMM) | 3161 | -2.79 |
| RAD51 | RAD51 homolog (RecA homolog, E. coli) (S. cerevisiae) | 5888 | -2.84 |
| DTL | RA-regulated nuclear matrix-associated protein | 51514 | -2.92 |
| CENPE | centromere protein E, 312kDa | 1062 | -2.93 |
| CCNE2 | cyclin E2 | 9134 | -2.97 |
| CNIH3 | cornichon homolog 3 (Drosophila) | 149111 | -3.03 |
| RAD51AP1 | RAD51 associated protein 1 | 10635 | -3.05 |
| WDHD1 | WD repeat and HMG-box DNA binding protein 1 | 11169 | -3.19 |
| DEPDC1 | DEP domain containing 1 | 55635 | -3.20 |
| BUB1 | BUB1 budding uninhibited by benzimidazoles 1 homolog (yeast) | 699 | -3.22 |
| C18orf24 | chromosome 18 open reading frame 24 | 220134 | -3.24 |
| FEN1 | flap structure-specific endonuclease 1 | 2237 | -3.27 |
| AOX1 | aldehyde oxidase 1 | 316 | -3.31 |
| MKI67 | antigen identified by monoclonal antibody Ki-67 | 4288 | -3.37 |
| PLK1 | polo-like kinase 1 (Drosophila) | 5347 | -3.58 |
| TTK | TTK protein kinase | 7272 | -3.63 |
| KIF4A | Kinesin family member 4A | 24137 | -3.65 |
| DCC1 | defective in sister chromatid cohesion homolog 1 (S. cerevisiae) | 79075 | -3.67 |
| SHCBP1 | SHC SH2-domain binding protein 1 | 79801 | -3.67 |
| DLG7 | discs, large homolog 7 (Drosophila) | 9787 | -3.77 |
| AURKB | aurora kinase B | 9212 | -3.90 |
| CCNA2 | cyclin A2 | 890 | -4.01 |
| GPSM2 | G-protein signalling modulator 2 (AGS3-like, C. elegans) | 29899 | -4.11 |
| BUB1B | BUB1 budding uninhibited by benzimidazoles 1 homolog beta (yeast) | 701 | -4.13 |
| KIF2C | kinesin family member 2C | 11004 | -4.25 |
| CDC25A | cell division cycle 25A | 993 | -4.50 |
| CDC2 | Cell division cycle 2, G1 to S and G2 to M | 983 | -4.95 |
| NEK2 | NIMA (never in mitosis gene a)-related kinase 2 | 4751 | -4.98 |
| CENPA | centromere protein A, 17kDa | 1058 | -5.09 |
| KIF23 | kinesin family member 23 | 9493 | -5.17 |
| KNTC2 | kinetochore associated 2 | 10403 | -6.03 |
| ARHGAP11A | Rho GTPase activating protein 11A | 9824 | -6.05 |
| ASPM | asp (abnormal spindle)-like, microcephaly associated (Drosophila) | 259266 | -6.71 |
| HAS1 | hyaluronan synthase 1 | 3036 | -7.15 |
| TIAM1 | T-cell lymphoma invasion and metastasis 1 | 7074 | -7.63 |
| ORC5 | origin recognition complex, subunit 5-like (yeast) | 5001 | -8.97 |
| TNFRSF10C | tumor necrosis factor receptor superfamily, member 10c, | 8794 | -10.93 |
| LGR5 | leucine-rich repeat-containing G protein-coupled receptor 5 | 8549 | -12.02 |
