# Supplementary material for: p21 as a Transcriptional Co-Repressor of S-Phase and Mitotic Control Genes
Source: PLoS One. 2012 May 25;7(5):e37759. doi: 10.1371/journal.pone.0037759 (PMC3360621; doi:10.1371/journal.pone.0037759)
Supplement: Table S3 — Genes regulated by p21 in primary keratinocytes. The table list the genes showing an expression change in human primary keratinocytes infected with adenovirus-p21 as described in Methods. RNA was analysed 24 h after infection. The 75 genes are included in the heat map of Fig. 7. The table includes genes with a fold change ≥log21.2 (≥2.3-fold) and with a signal difference ≥50 between both experimental conditions (as defined by dChip program and with Affymetrix U133 biochip data). Values are mean of fold changes (expressed as log2) of two independent experiments (p<0.001). For those genes represented by two or three Affymetrix probes, the fold change is the mean between the values of the probes. A negative fold change indicates down-regulation upon p21 induction. (DOC) [file pone.0037759.s007.doc]

**Ferrandiz et al.**

**p21 as a transcriptional co-repressor of S-phase and mitotic control genes**

**Supporting Information**

**Table S3. Genes regulated by p21 in primary keratinocytes**. The table list the genes showing an expression change in human primary keratinocytes infected with adenovirus-p21 as described in Methods. RNA was analysed 24 h after infection. The 75 genes are included in the heat map of Fig. 7. The table includes genes with a fold change ≥log_2_1.2 (≥2.3-fold) and with a signal difference ≥50 between both experimental conditions (as defined by dChip program and with Affymetrix U133 biochip data). Values are mean of fold changes (expressed as log_2_) of two independent experiments (p<0.001). For those genes represented by two or three Affymetrix probes, the fold change is the mean between the values of the probes. A negative fold change indicates down-regulation upon p21 induction.

| **Symbol** | **Gene Description** | **Gene ID** | **Fold change (log2)** |
| --- | --- | --- | --- |
| NEK1 | NIMA (never in mitosis gene a)-related kinase 1 | 4750 | -0.87 |
| CDC16 | cell division cycle 16 homolog (S. cerevisiae) | 8881 | -0.95 |
| CCNG1 | cyclin G1 | 900 | -1.01 |
| CDC25C | cell division cycle 25C | 995 | -1.04 |
| PPP1CC | protein phosphatase 1, catalytic subunit, gamma isoform | 5501 | -1.04 |
| CDC14B | CDC14 cell division cycle 14 homolog B (S. cerevisiae) | 8555 | -1.05 |
| BIRC5 | baculoviral IAP repeat-containing 5 | 332 | -1.06 |
| CCNI | cyclin I | 10983 | -1.06 |
| PPP2R4 | protein phosphatase 2A, regulatory subunit B' (PR 53) | 5524 | -1.07 |
| PPM1G | protein phosphatase 1G magnesium-dependent, gamma isoform | 5496 | -1.07 |
| RINT1 | RAD50 interactor 1 | 60561 | -1.08 |
| CCND3 | cyclin D3 | 896 | -1.09 |
| CDC42EP4 | CDC42 effector protein (Rho GTPase binding) 4 | 23580 | -1.09 |
| CNNM1 | cyclin M1 | 26507 | -1.11 |
| NEK7 | NIMA (never in mitosis gene a)-related kinase 7 | 140609 | -1.12 |
| WEE1 | Wee1homolog (S. pombe) | 7465 | -1.12 |
| CDK6 | cyclin-dependent kinase 6 | 1021 | -1.12 |
| KIFAP3 | kinesin-associated protein 3 | 22920 | -1.12 |
| RAD50 | RAD50 homolog (S. cerevisiae) | 10111 | -1.12 |
| PAK2 | p21 (CDKN1A)-activated kinase 2 | 5062 | -1.12 |
| KIF3C | kinesin family member 3C | 3797 | -1.13 |
| RAD21 | RAD21 homolog (S. pombe) | 5885 | -1.13 |
| DMTF11 | cyclin D binding myb-like transcription factor 1 | 9988 | -1.13 |
| CCNA1 | cyclin A1 | 8900 | -1.14 |
| PPP1CA | protein phosphatase 1, catalytic subunit, alpha isoform | 5499 | -1.14 |
| RCC1 | regulator of chromosome condensation 1 | 1104 | -1.14 |
| BIRC4 | baculoviral IAP repeat-containing 4 | 331 | -1.14 |
| NEK4 | NIMA (never in mitosis gene a)-related kinase 4 | 6787 | -1.14 |
| PPP3CA | protein phosphatase 3, catalytic subunit, alpha isoform (calcineurin A alpha) | 5533 | -1.15 |
| CDK7 | cyclin-dependent kinase 7 | 1022 | -1.15 |
| PPP1R14B | protein phosphatase 1, regulatory (inhibitor) subunit 14B | 26472 | -1.15 |
| RAD23B | RAD23 homolog B (S. cerevisiae) | 5887 | -1.15 |
| KIF5C | kinesin family member 5C | 3800 | -1.15 |
| ORC4 | origin recognition complex, subunit 4-like (yeast) | 5000 | -1.15 |
| CDK2AP1 | CDK2-associated protein 1 | 8099 | -1.15 |
| STK24 | serine/threonine kinase 24 (STE20 homolog, yeast) | 8428 | -1.15 |
| CDKN2D | cyclin-dependent kinase inhibitor 2D (p19, inhibits CDK4) | 1032 | -1.15 |
| CCNE2 | cyclin E2 | 9134 | -1.15 |
| CDK5R1 | cyclin-dependent kinase 5, regulatory subunit 1 (p35) | 8851 | -1.15 |
| CDC14A | CDC14 cell division cycle 14 homolog A (S. cerevisiae) | 8556 | -1.15 |
| CCND2 | cyclin D2 | 894 | -1.15 |
| CDKN2B | cyclin-dependent kinase inhibitor 2B (p15) | 1030 | -1.15 |
| PPP2R1A | protein phosphatase 2 regulatory subunit A (PR 65), alpha isoform | 5518 | -1.15 |
| PPP3CC | protein phosphatase 3, catalytic subunit, gamma isoform (calcineurin A gamma) | 5533 | -1.15 |
| AURKB | aurora kinase B/serine threonine kinase 5 | 9212 | -1.15 |
| CCND1 | cyclin D1 | 595 | -1.15 |
| CDK9 | cyclin-dependent kinase 9 (CDC2-related kinase) | 1025 | -1.15 |
| CDC37 | CDC37 cell division cycle 37 homolog (S. cerevisiae) | 11140 | -1.15 |
| PPP1R7 | protein phosphatase 1, regulatory subunit 7 | 5510 | -1.15 |
| PAK6 | p21(CDKN1A)-activated kinase 6 | 56924 | -1.15 |
| KIF2 | Kinesin heavy chain member 2 | 3796 | -1.15 |
| PPEF2 | protein phosphatase, EF-hand calcium binding domain 2 | 5470 | 0.65 |
| PPM1A | protein phosphatase 1A (formerly 2C) | 5494 | 0.81 |
| PPP2R1B | protein phosphatase 2, regulatory subunit A (PR 65), beta isoform | 5519 | 0.87 |
| PPP1R1A | protein phosphatase 1, regulatory (inhibitor) subunit 1A | 5502 | 0.89 |
| CDKN2C | cyclin-dependent kinase inhibitor 2C (p18, inhibits CDK4) | 1031 | 0.91 |
| RAD1 | RAD1 homolog (S. pombe) | 5810 | 0.99 |
| STK3 | serine/threonine kinase 3 (STE20 homolog, yeast) | 6788 | 1.03 |
| STK4 | serine/threonine kinase 4 | 6789 | 1.04 |
| NEK11 | NIMA (never in mitosis gene a)-related kinase 11 | 79858 | 1.06 |
| PPP2R2B | protein phosphatase 2 (formerly 2A), regulatory subunit B (PR 52), beta isoform | 5521 | 1.06 |
| KIF1C | kinesin family member 1C | 10749 | 1.07 |
| GAK | cyclin G associated kinase | 2580 | 1.07 |
| TNK2 | tyrosine kinase, non-receptor, 2 | 10188 | 1.09 |
| DUSP13 | dual specificity phosphatase 13 | 51207 | 1.09 |
| CDKN2A | cyclin-dependent kinase inhibitor 2A (melanoma, p16, inhibits CDK4) | 1029 | 1.1 |
| KIF17 | kinesin family member 17 | 57576 | 1.11 |
| TBC1D1 | TBC1 (tre-2/USP6, BUB2, cdc16) domain family, member 1 | 23216 | 1.12 |
| CDC2L1 | cell division cycle 2-like 1 (PITSLRE proteins) | 984 | 1.12 |
| PPP2R5B | protein phosphatase 2, regulatory subunit B (B56), beta isoform | 5526 | 1.12 |
| CDC42 | cell division cycle 42 (GTP binding protein, 25kDa) | 998 | 1.13 |
| RAD54L | RAD54-like (S. cerevisiae) | 8438 | 1.13 |
| KIFC3 | kinesin family member C3 | 3801 | 1.13 |
| HERC5 | hect domain and RLD 5 | 51191 | 1.13 |
| CDKN1A | cyclin-dependent kinase inhibitor 1A (p21, Cip1) | 1026 | 1.14 |
| BUB1 | BUB1 budding uninhibited by benzimidazoles 1 homolog (yeast) | 699 | 1.15 |
| CCNT2 | cyclin T2 | 905 | 1.15 |
| CDKL3 | cyclin-dependent kinase-like 3 | 51265 | 1.15 |
| CDK5RAP1 | CDK5 regulatory subunit associated protein 1 | 51654 | 1.15 |
| PPP1R2 | protein phosphatase 1, regulatory (inhibitor) subunit 2 | 5504 | 1.15 |
| PPEF1 | protein phosphatase, EF-hand calcium binding domain 1 | 5475 | 1.15 |
| CNNM2 | cyclin M2 | 54805 | 1.15 |
